# Diversifying T-cell responses: safeguarding against pandemic influenza with mosaic nucleoprotein

**DOI:** 10.1128/jvi.00867-24

**Published:** 2025-02-03

**Authors:** Hongtae Park, Brock Kingstad-Bakke, Thomas Cleven, Myunghwan Jung, Yoshihiro Kawaoka, M. Suresh

**Affiliations:** 1 Department of Pathobiological Sciences, University of Wisconsin 5228 , Madison, Wisconsin, USA; St. Jude Children's Research Hospital, Memphis, Tennessee, USA

**Keywords:** influenza, T cell-based vaccines, nucleoprotein, mosaic, mucosal immunity

## Abstract

Pre-existing T-cell responses have been linked to reduced disease severity and better clinical outcomes during the 2009 influenza pandemic and the recent COVID-19 pandemic. We hypothesized that diversifying T-cell responses, particularly targeting conserved viral proteins such as the influenza A virus (IAV) nucleoprotein (NP), could protect against both epidemic and pandemic IAV strains. To test this, we created a mosaic nucleoprotein (MNP) by synthesizing a sequence that maximized the representation of 9-mer epitopes from 7422 NP sequences across human, swine, and avian IAVs. Notably, the MNP sequence showed high homology with the NP of the H5N1 strain affecting dairy cows in the ongoing outbreak. Mucosal immunization with the adjuvanted MNP vaccine induced robust CD8 and CD4 T-cell responses against both known immunodominant and *in silico* predicted subdominant epitopes. MNP-vaccinated mice challenged with epidemic H1N1 and H3N2 strains, which shared immunodominant CD8 and/or CD4 T-cell epitopes, showed a significant (~4 log) reduction in lung viral load. Importantly, MNP-vaccinated mice challenged with a pandemic H1N1 strain lacking shared immunodominant CD8 or CD4 epitopes exhibited a superior reduction in lung viral load, linked to T-cell responses targeting subdominant epitopes present in both the MNP and pandemic strain NP. These results suggest that a diversified T-cell response induced by the MNP vaccine could provide broad protection against severe disease from both current and emerging IAV strains.

**IMPORTANCE:**

The World Health Organization (WHO) estimates that seasonal influenza causes 3–5 million cases of severe illness annually. The influenza virus frequently undergoes genetic changes through antigenic drift and antigenic shift, resulting in annual epidemics and occasional pandemics. Consequently, a major public health objective is to develop a universal influenza vaccine that offers broad protection against both current and pandemic influenza A strains. In this study, we designed a nucleoprotein (NP) antigen (termed mosaic NP) comprising antigenic regions found in thousands of influenza viruses, aiming to use it as a vaccine to induce broad anti-influenza T-cell responses. Our findings indicate that the mosaic NP vaccine provided significant protection against seasonal H1N1 and H3N2, as well as the pandemic H1N1 strain, demonstrating its effectiveness across various influenza subtypes. These findings suggest that the mosaic NP is a potential universal influenza vaccine antigen, capable of protecting against diverse strains of influenza viruses.

## INTRODUCTION

The influenza virus presents a significant global health threat, with mutations in its hemagglutinin surface glycoprotein leading to antigenic drifts and the continual generation of new variants, precipitating annual influenza epidemics worldwide (1, 2). Current antibody-based vaccines primarily target the variable hemagglutinin (HA) surface glycoprotein, but they exhibit reduced effectiveness against antigenically distinct drifted subtypes of influenza A viruses (IAVs), highlighting the need for alternative approaches (3–5). Notably, T-cell responses, particularly those targeting internal proteins, demonstrate broad reactivity across various IAV subtypes (6, 7). Evidence suggests that pre-existing cross-reactive T-cell responses were protective against severe disease during the 2009 swine influenza pandemic and also expedited recovery in individuals infected with H7N9 and other IAV strains (8–11). This has sparked considerable interest in harnessing T-cell immunity to develop universal vaccines capable of providing cross-protection against diverse influenza strains, representing a promising avenue in the ongoing battle against the virus.

There are multiple approaches to the design of broadly reactive vaccines to address the challenge of pathogen diversity. One is to focus on conserved antigens, those parts of the pathogen least likely to mutate over time, such as influenza nucleoprotein (NP). Another approach is to formulate multivalent vaccines that include several variants of the same protein, such as the trivalent influenza vaccine. Both approaches still fall short of capturing maximum epitope diversity, and therefore another strategy is to specifically aim for centralized antigens, either by phylogenetic identification of an evolutionally central strain or by algorithmically averaging, where sequences from multiple variants of the same protein are combined into a single synthetic construct, typically as a consensus sequence. Mosaic protein sequences are an alternative approach to consensus sequence design and are crafted through a computational process that incorporates epitopes from diverse viral strains, into a single synthetic antigen (12, 13). This contrasts with consensus sequences, which are constructed by averaging sequences across variants to form a generalized, representative profile (14). Mosaic proteins are designed using algorithms that optimize epitope coverage to ensure broad reactivity against multiple IAV strains. Such methodologies aim to maintain epitopes that are both common and rare across strains, maximizing the immune system’s ability to recognize different viruses (15). Prior studies have demonstrated the superiority of mosaic over consensus sequence approaches in vaccine design, particularly in their capacity to elicit a wider breadth of immune responses. This was notably demonstrated in the field of HIV vaccines, where mosaic antigens provided superior coverage against global HIV isolates compared to consensus designs (16). The broad protective potential of mosaic-based vaccines has also been validated in influenza research. Prior work has shown that mosaic hemagglutinin constructs conferred protection against a wide spectrum of influenza subtypes in various species, reflecting a significant advance toward a universal influenza vaccine. This included effective protection in mice, chickens, and monkeys against both seasonal and highly pathogenic avian influenza strains (17–20). Considering the highly conserved nature of the nucleoprotein (NP) in influenza viruses, the application of mosaic technology to NP could be particularly advantageous.

In this study, we designed mosaic NP (MNP) based on all available sequences derived from human, avian, and swine IAV strains. The resulting MNP was tested for T-cell-mediated viral control when administered with vaccine adjuvants known to induce robust T-cell immune responses. Compared to the recombinant NP (PR8-NP) derived from the H1N1 Influenza A/PR/8/34 (IAV-PR8) strain, the MNP elicited CD4 T-cell responses against a broader pool of epitopes in mice and demonstrated superior protective efficacy against pandemic IAV, as compared to PR8 NP.

## RESULTS

### Characterization of mosaic NP

We designed a single influenza virus nucleoprotein (termed mosaic NP, consisting of amino acids that are naturally present in the influenza virus nucleoproteins from different viral strains), which incorporates the maximum number of 9-mer sequences derived from 7422 NP sequences in the database (designed using the Mosaic Vaccine tool available at hiv.lanl.gov.). The resulting 498-amino-acid (AA) MNP was then analyzed for its coverage across all possible 9-mer epitopes found in the entire set of 7422 NP sequences. Remarkably, greater than 77% of all possible NP 9-mers were covered by the MNP (Fig. S1A). Coverage of all epitopes was nearly complete when 9-mer epitopes are only off by 2 (Table 1) or fewer amino acids; 2 AA mismatched coverage of all NP 9-mer epitopes by MNP was greater than for sample natural sequences. While the MNP sequence was designed for optimal 9-mer coverage, we additionally evaluated for 12-mer and 14-mer coverage attained against natural NP sequences and found that the exact coverage of unique epitopes within the pool of 7422 sequences was approximately 19%–27% higher, as compared to the natural NP sequences of epidemic and pandemic IAV strains (Table 1).

**TABLE 1:** Coverage of unique 9-mer, 12-mer, or 14-mer from the 7,422 sequences used to design the MNP, by the mosaic sequence vs reference influenza strains

NP/strain	Exact	Off by 1	Off by 2
9-mer			
Mosaic NP	0.7714	0.9395	0.9841
H3N2 A/Aichi/2/1968	0.4542	0.7250	0.8198
H1N1 A/PR/8/34	0.5385	0.8550	0.9631
H1N1 A/CA/04/09	0.6044	0.8620	0.9621
12-mer			
Mosaic NP	0.7015	0.9113	0.9710
H3N2 A/Aichi/2/1968	0.4314	0.7535	0.8967
H1N1 A/PR/8/34	0.4318	0.7846	0.9347
H1N1 A/CA/04/09	0.5169	0.7988	0.9303
14-mer			
Mosaic NP	0.6645	0.8906	0.9615
H3N2 A/Aichi/2/1968	0.3788	0.6939	0.8673
H1N1 A/PR/8/34	0.3659	0.7354	0.9136
H1N1 A/CA/04/09	0.4651	0.7576	0.9052

**Fig 1:**
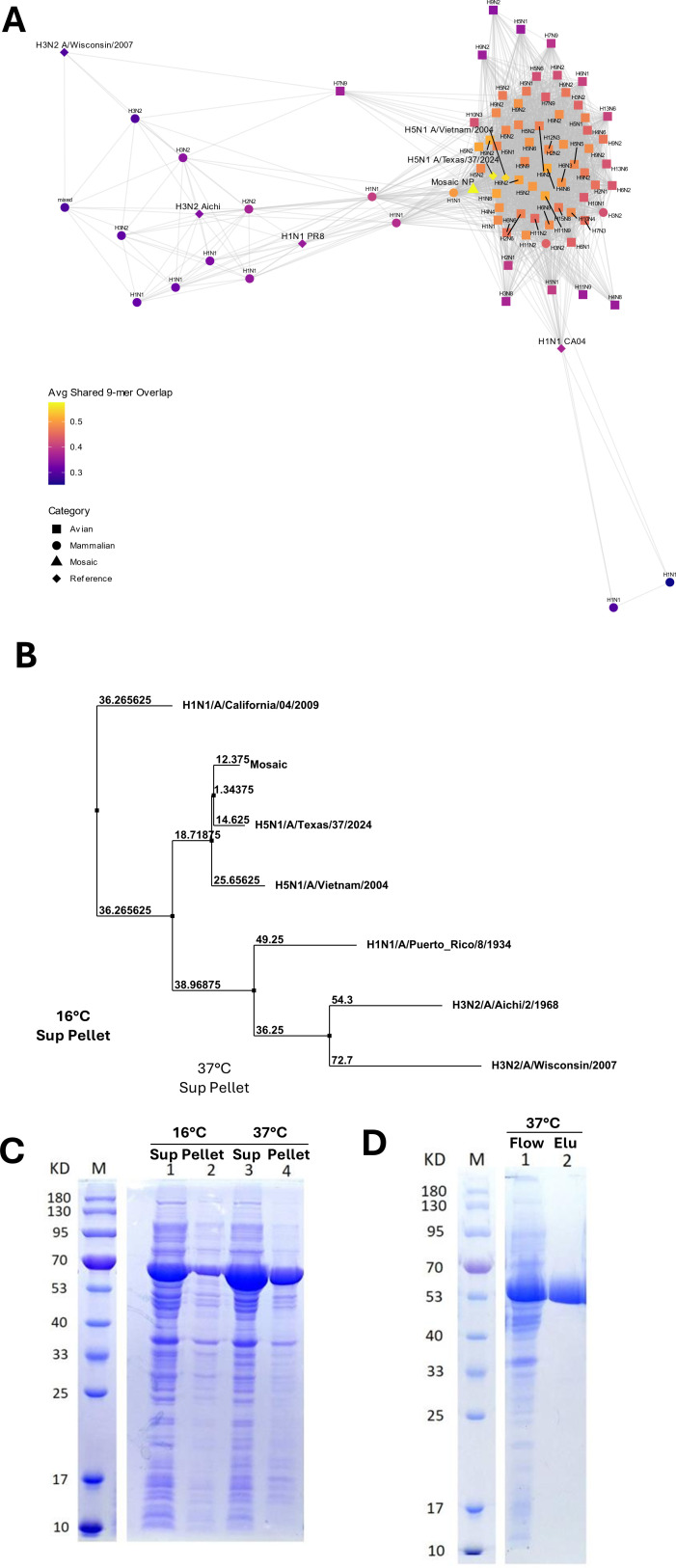
Characterization and synthesis of mosaic NP. (**A**) Influenza sequences were identified by extracting unique 9-mers from the mosaic-derived 7422 NP amino acid sequences and calculating Jaccard similarities between the sequences to assess shared motifs. A network graph was constructed, where nodes represent NP sequences and edges indicate significant 9-mer overlap. Mammalian and avian hosts were differentiated by distinct node shapes, and the Mosaic NP, and reference sequences were highlighted. The average shared 9-mer overlap is indicated by node colors. (**B**) The phylogenetic distance between the aligned sequences was calculated and visualized using the neighbor-joining method based on the BLOSUM62 scoring. (**C**) SDS-PAGE analysis of mosaic protein expression in sonicated *E. coli* cells following induction at either 16°C or 37°C. Lane M: protein marker. Lanes 1 and 2: supernatant and pellet from 16°C induction, respectively. Lanes 3 and 4: supernatant and pellet from 37°C induction, respectively. (**D**) SDS-PAGE analysis of HIS-tag purification from supernatants of sonicated cells induced at 37°C induced. Lane M: protein marker. Lane 1: flow through. Lane 2: elution.

To demonstrate the capacity of the MNP to capture sequence diversity among both avian and mammalian influenza NP genes, we generated a network graph (Fig. 1A) that visualizes the degree of 9-mer overlap between representative influenza sequences. Avian sequences form a densely connected cluster, distinctly separate from a more dispersed group of mammalian sequences. The synthetic Mosaic NP sequence is centrally located between these clusters, exhibiting extensive shared NP 9-mer overlaps with both avian and mammalian sequences, as evidenced by numerous connecting edges, and high average 9-mer overlap. Furthermore, AA sequence comparison between the MNP and the six representative IAV strains including the recent bovine epidemic H5N1 strain (two of H1N1, two of H3N2, and two of H5N1) resulted in the consensus AA sequences of NP and there were only five amino acid differences between the generated consensus sequence and the MNP (Fig. S1B). It is noteworthy that the MNP exhibited the highest similarity with the newly reported highly pathogenic avian influenza (HPAI) H5N1 strain that infected cattle in 2024, which was not present in the database used for the generation of MNP (Fig. 1B). The recombinant protein generated from this MNP sequence was successfully expressed in BL21 (DE3) *E. coli* and purified using Ni-NTA affinity chromatography (Fig. 1C and D).

### Mosaic nucleoprotein elicits potent T-cell responses and protects against lethal challenge with H1N1 influenza A virus

**Fig 2:**
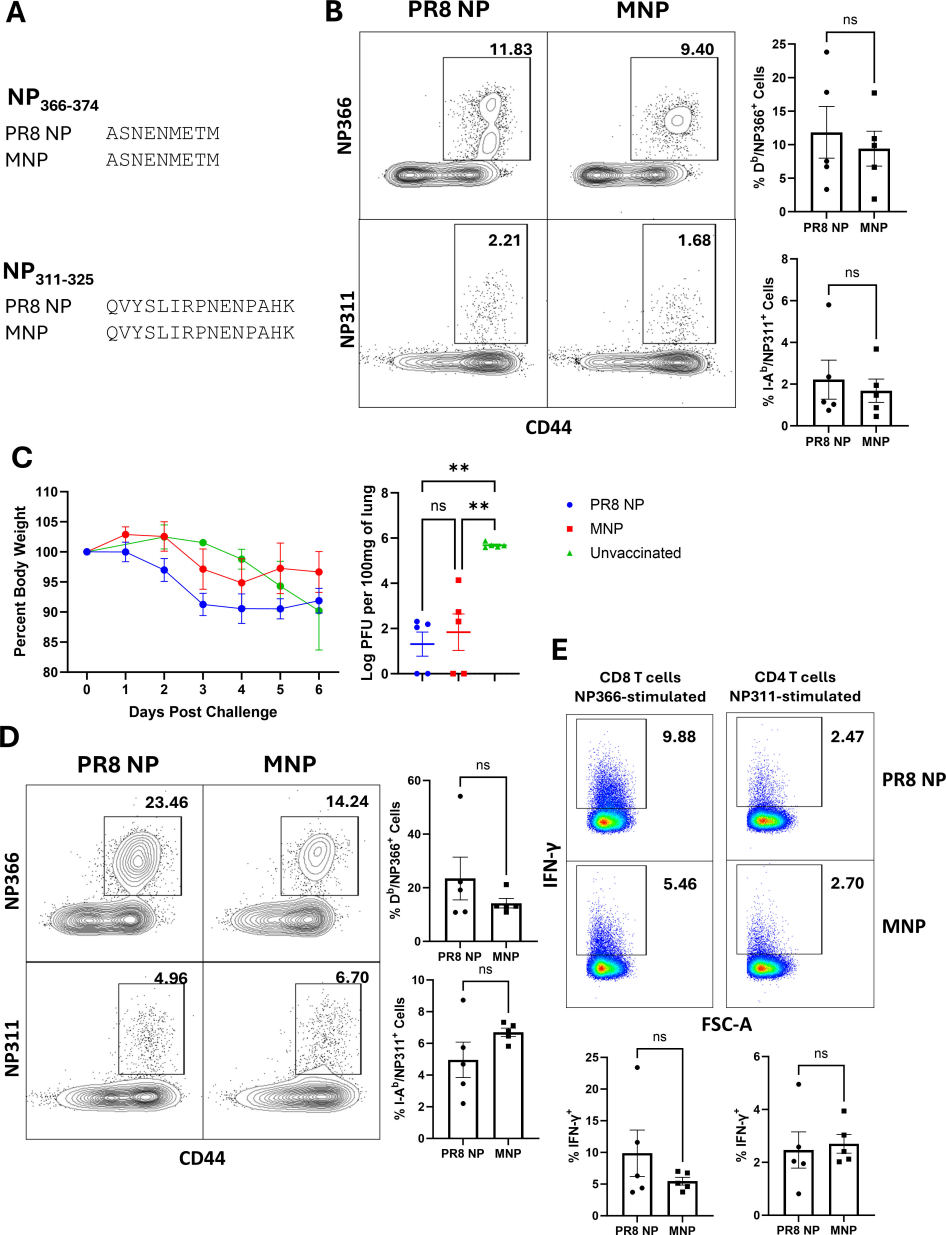
Mosaic and PR8 NP vaccine-induced reduction in homologous Influenza A viral titers in lungs. Cohorts of 7- to 12-week-old mice were vaccinated intranasally twice with either mosaic NP (MNP) or PR8 NP formulated in ADJ + GLA. (**A**) Sequence comparisons between the PR8 NP and the MNP were performed for the regions NP311-325 and NP366-374, which are immunodominant epitopes for the PR8 NP. (**B**) By staining with MHC I or MHC II tetramers, NP311- and NP366-specific CD4 and CD8 T cells were quantified at 8 days after booster vaccination. (**C–E**) At 120 days after booster vaccination, mice were challenged with the IAV-PR8 virus. (**C**) Six days after the challenge, viral titer was quantified in the lungs and weight loss was monitored. (**D**) Using MHC tetramers, NP311- and NP366-specific CD4 and CD8 T cells were quantified in the lungs on the 6th day after the challenge. (**E**) Lung NP366- and NP311-specific T cells producing IFN-γ were quantified by *ex vivo* stimulation with the respective peptides. Planned pairwise group comparisons were conducted using Welch’s ANOVA followed by Welch’s t-tests, or an unpaired t test for two-group comparisons. *, **, ***, and **** denote significance at *P* < 0.05, 0.01, 0.001, and 0.0001, respectively. "ns" indicates results that are “not significant.” The data in each graph represent the mean ± SEM. Data are representative of at least two independent experiments. The FACS plots generated from concatenated data from all mice in the respective group show the percentages of cells within the gate among CD8 or CD4 T cells, which correspond to the average values across individual samples.

To assess whether MNP is immunogenic, we formulated the protein in a combination adjuvant consisting of Adjuplex (ADJ; carbomer-based adjuvant) and glucopyranosyl lipid A (GLA; TLR-4 agonist), as described previously (21). For rigor, the PR8 NP from the IAV-PR8 strain was included as a positive control, and mice were vaccinated twice intranasally, at an interval of 3 weeks. It is noteworthy that the immunodominant D^b^-restricted NP366 and I-A^b^-restricted NP311 epitopes in the PR8 NP are conserved in the MNP (Fig. 2A). On the 8th day after vaccination, we quantified NP366- and NP311-specific T-cell responses in lungs. In the lungs of vaccinated mice, approximately 10% of lung tissue CD8 T cells were specific to NP366 epitope, and ~2% of CD4 T cells were specific to the NP311-epitope (Fig. 2B). The percentages of NP366- or NP311-specific T cells in lungs of PR8 NP and MNP groups were similar, which suggested that the MNP is as immunogenic as the native PR8 NP protein for these immune dominant epitopes.

Since MNP elicited immunodominant epitope-specific effector CD4 and CD8 T cells at levels comparable to that of PR8 NP, we assessed viral protection by challenging vaccinated and unvaccinated mice with the pathogenic IAV-PR8 strain. At 120 days after vaccination, mice were challenged intranasally with a lethal dose of IAV-PR8. Remarkably, the lungs of mice vaccinated with the PR8 NP or the MNP contained approximately 4 logs lower viral titers, as compared to lungs from unvaccinated mice. Consistent with accelerated virus control, recovery from weight loss in vaccinated mice started from the 4th day after the viral challenge (Fig. 2C). Next, we examined whether viral control in the lungs of MNP-vaccinated mice was linked to strong recall responses of vaccine-induced memory T cells. On the 6th day after viral challenge, we detected high percentages of NP366-specific CD8 T cells and NP311-specific CD4 T cells in lungs of both PR8 NP- and MNP-vaccinated mice (Fig. 2D). Furthermore, NP366-specific CD8 T cells and NP311-specific CD4 T cells readily produced IFN-γ, upon *ex vivo* antigenic stimulation (Fig. 2E). Taken together, data in Fig. 2 strongly suggested that (i) MNP is appropriately processed by the antigen presentation machinery to elicit NP366-specific and NP 311-specific effector and memory T cells; (ii) memory T cells induced by MNP are appropriately programmed for swift recall responses and effectively reduce influenza viral load in lungs; and (iii) conserved immunodominant epitopes are sufficient for T-cell-dependent viral control to IAV.

### T-cell responses to conserved immunodominant epitopes in the mosaic nucleoprotein protect against the H3N2 influenza A virus

**Fig 3:**
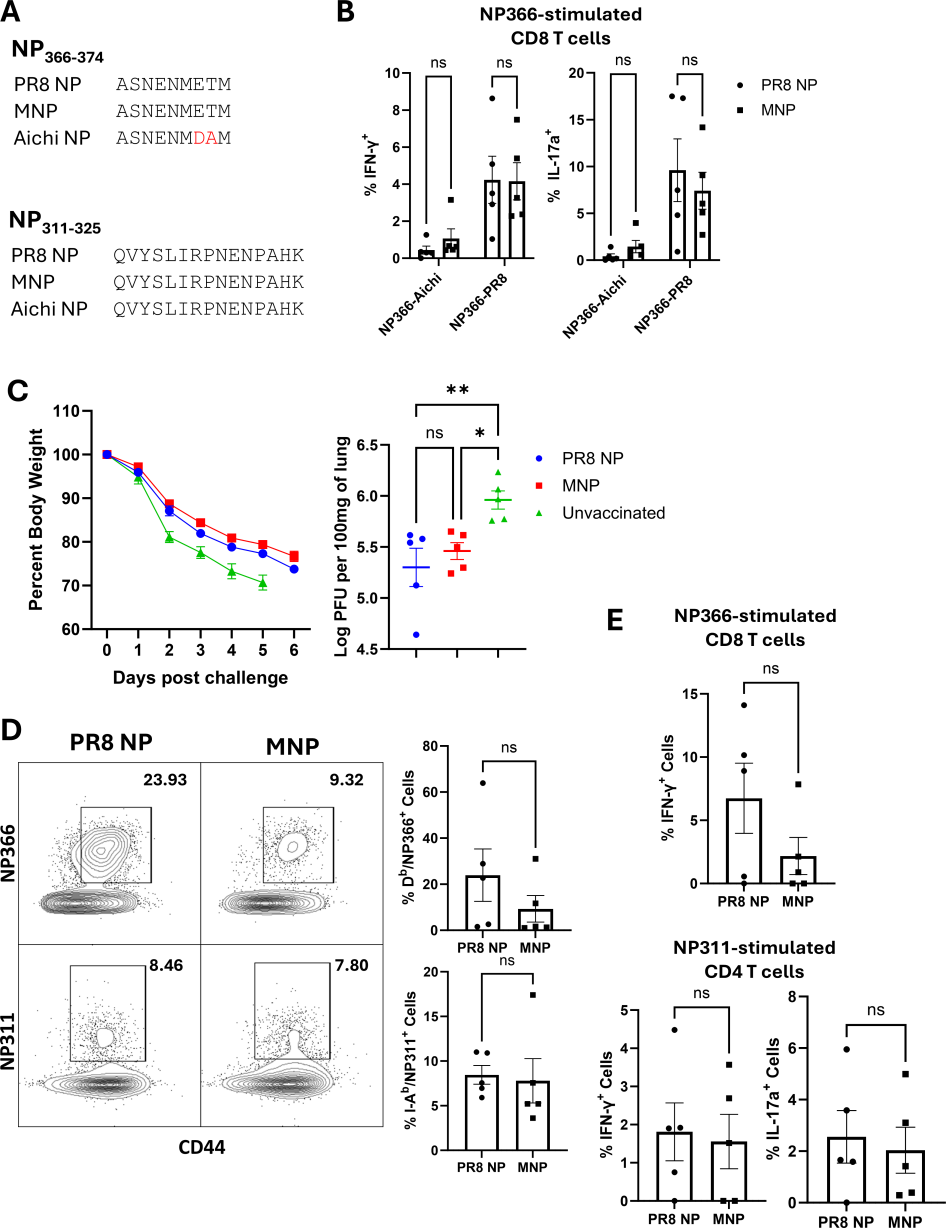
Mosaic and PR8 NP vaccine-induced reduction in H3N2 influenza A viral titers in lungs. Cohorts of 7- to 12-week-old mice were vaccinated intranasally twice with either MNP or PR8 NP formulated in the combination adjuvant ADJ + GLA. At 6 months after booster vaccination, mice were challenged with the H3N2 Influenza A/Aichi/2/1968 strain; unvaccinated mice were challenged as controls. (**A**) Sequence comparisons were performed between PR8 NP, MNP, and Aichi NP for the two immunodominant epitopes NP311-325 and NP366-374. (**B**) Lung CD8 T cells producing IFN-γ and/or IL-17 were quantified by *ex vivo* stimulation with the Aichi-NP366 or the PR8 NP366 peptide on day eight after booster vaccination. (**C**) Six days after the challenge, viral titer was quantified in the lungs and weight loss was monitored. (**D**) Using MHC tetramers, NP311- and NP366-specific CD4 and CD8 T cells were quantified in the lungs on the 6th day after the challenge. (**E**) Lung NP366- and NP311-specific T cells producing IFN-γ and/or IL-17 were quantified by *ex vivo* stimulation with the respective peptides. Planned pairwise group comparisons were conducted using a one-way ordinary ANOVA followed by Fisher’s least-significant difference (LSD) test, or an unpaired t test was used for two-group comparisons. *, **, ***, and **** denote significance at *P* < 0.05, 0.01, 0.001, and 0.0001, respectively. "ns" indicates results that are “not significant.” The data in each graph represent the mean ± SEM. The FACS plots generated from concatenated data from all mice in the respective group show the percentages of cells within the gate among CD8 or CD4 T cells, which correspond to the average values across individual samples.

Data in Fig. 2 showed that mosaic NP formulated in ADJ + GLA elicited T-cell memory to immunodominant epitopes and protected against the H1N1 influenza A virus. Next, we compared the amino acid sequence of MNP with the NP of the H3N2 Influenza A/Aichi/2/1968 strain (IAV-Aichi). While the MHC II-restricted NP311 epitope in the MNP and IAV-Aichi NP are identical, the MHC I-restricted NP366 epitope of IAV-Aichi differs from the MNP epitope in two amino acids at positions 7 and 8 of the sequence (Fig. 3A). To assess whether the two amino acid difference in the NP366 epitope in the IAV-Aichi virus was sufficient to evade T-cell immunity induced by the MNP, cross-reactivity between the two epitopes was measured after vaccination, and viral titers were assessed following the challenge. We vaccinated cohorts of mice with PR8 NP or the MNP. Eight days after vaccination, lung T cells were stimulated with the Aichi-NP366 ASNENMDAM peptide, and cytokine expression, including IFN-γ and IL-17a, was measured (Fig. 3B). Results showed that PR8- or MNP-induced NP366-specific CD8 T cells were responsive to NP366-Aichi peptide stimulation, but the percentages of responding cells were lower, as compared to the percentages of cells that responded to the NP366-PR8 peptide. Six months after vaccination, we challenged vaccinated and unvaccinated mice with IAV-Aichi, and lung viral titers were quantified on the 6th day after the challenge. Results showed sustained weight loss in all three groups, but both vaccinated groups exhibited significant reductions in viral titers, as compared to the unvaccinated control group (Fig. 3C). We determined whether lower lung viral burden in vaccinated groups was associated with robust recall CD8 and/or CD4 T-cell responses. Mismatch of two amino acid residues in the NP366 epitope between the IAV-Aichi NP and the MNP or PR8 NP led to substantive variability in the magnitude of the NP366-specific recall CD8 T-cell responses in lungs of vaccinated mice (Fig. 3D and E). These results, along with the data on the lower T-cell response to the vaccine (Fig. 3B), suggested incomplete cross-reactivity, which may also explain the relatively higher lung IAV-Aichi viral titers, as compared to the lungs of MNP-vaccinated mice following PR8 challenge. However, consistent with the conservation of the NP311 epitope in PR8 NP, MNP, and the IAV-Aichi NP, NP311-specific recall CD4 T-cell responses were less variable and uniformly strong in both vaccinated groups (Fig. 3D and E). In response to *ex vivo* stimulation with the respective peptides, lung NP366- and NP311-specific T cells produced IFN-γ and/or IL-17. Thus, despite high variability in the NP366-specific response, strong and consistent CD4 T-cell response was sufficient to achieve effective viral control in the lungs of MNP-vaccinated mice.

### Heterosubtypic T-cell immunity to pandemic IAV H1N1-2009 strain

**Fig 4:**
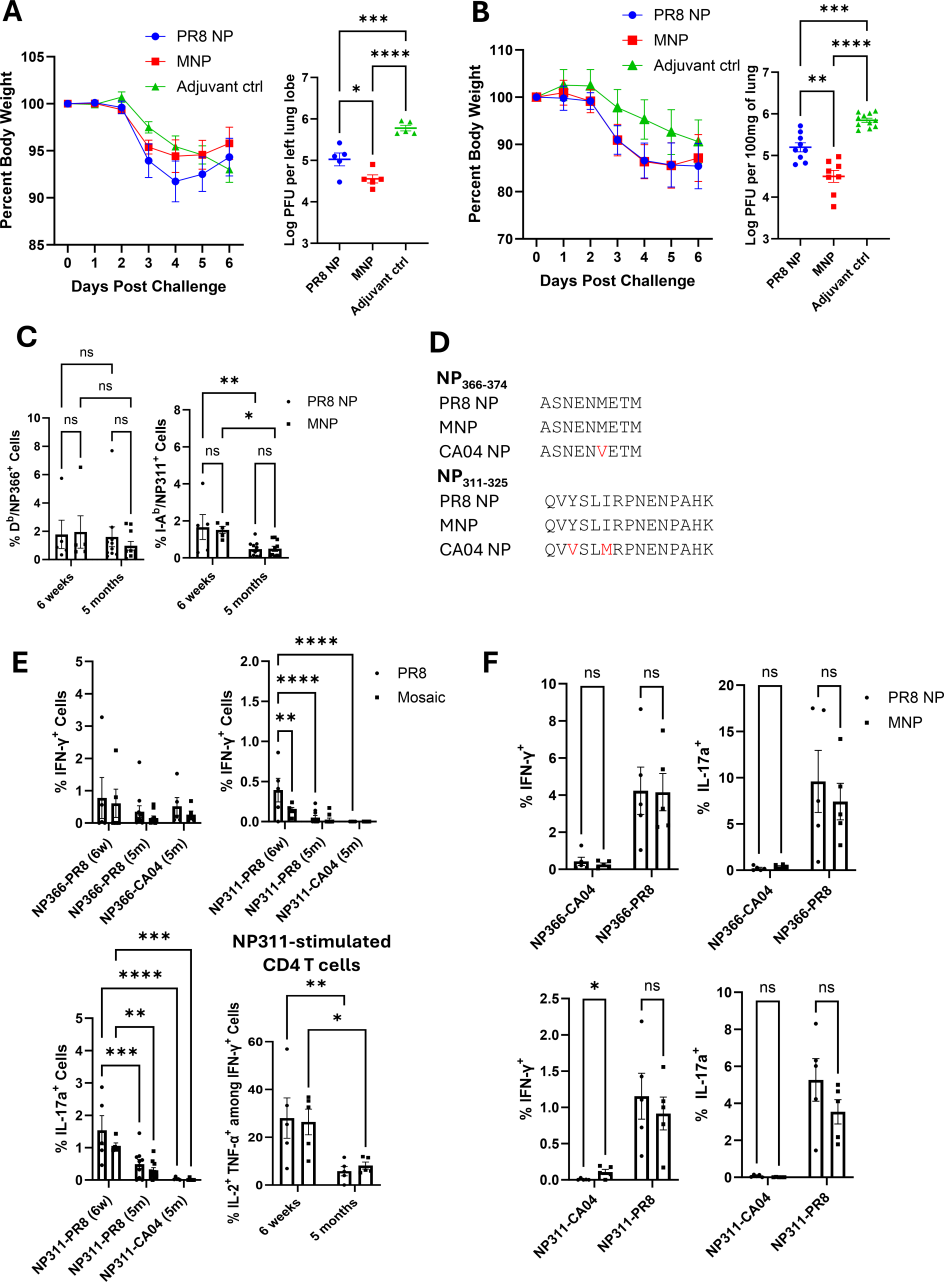
Mosaic and PR8 NP vaccine-induced reduction in pandemic H1N1 influenza A virus titers in the lungs. Cohorts of 7- to 12-week-old mice were vaccinated intranasally twice with either MNP or PR8 NP formulated in the combination adjuvant ADJ + GLA. At 6 weeks or 5 months after booster vaccination, mice were challenged with the H1N1 Influenza A/California/04/2009 strain. Mice that were given only adjuvant were used as controls for the challenge. (**A**) Percent weight loss and viral titer in the lungs were assessed on the 6th day after the challenge in mice at 6 weeks post-booster vaccination. (**B**) Percent weight loss and viral titer in the lungs were assessed on the 6th day after the challenge in mice at 5 months post-booster vaccination. (**C**) By staining with MHC tetramers NP311- and NP366-specific CD4 and CD8 T cells were quantified in the lungs on the 6th day after the challenge. (**D**) Sequence comparisons were conducted among PR8 NP, MNP, and CA04 NP for the NP311-325 and NP366-374 regions. (**E**) At day 6 after viral challenge, lung NP366- and NP311-specific T cells producing IFN-γ, IL-2, TNF-α, and/or IL-17 were quantified by *ex vivo* stimulation with the respective peptides. (**F**) Lung CD8 and CD4 T cells producing IFN-γ and/or IL-17 were quantified by *ex vivo* stimulation with the CA04-NP366 or CA04-NP311 peptide, respectively, on day 8 after booster vaccination. Planned pairwise group comparisons were made using either ordinary one-way or two-way ANOVA with multiple comparisons using Fisher’s LSD test. *, **, ***, and **** denote significance at *P* < 0.05, 0.01, 0.001, and 0.0001, respectively. "ns" indicates results that are “not significant.” The data in each graph represent the mean ± SEM. Data are from three independent experiments.

The presence of pre-existing T-cell responses has been associated with less severe influenza disease in humans during the H1N1 pandemic of 2009 and improved recovery following H7N9 infections (8–11). Here, we investigated whether the collection of epitopes in the MNP can protect against the pandemic Influenza A/California/04/2009 (IAV-CA04) strain. Mice were vaccinated with the MNP or the H1N1 PR8 NP and challenged with the IAV-CA04 virus at 6 weeks after vaccination. While control mice displayed continuous weight loss following the viral challenge, mice vaccinated with PR8 NP, or the MNP showed recovery beginning on day 4 after the viral challenge (Fig. 4A). The lungs from both PR8 NP and MNP mice contained lower viral burden, as compared to controls. Notably, viral load in the lungs of mice vaccinated with MNP was significantly lower (*P* < 0.05) than in the lungs of PR8 NP mice (Fig. 4A). MNP-vaccinated mice showed enhanced viral control when challenged with IAV-CA04 even 5 months after vaccination (Fig. 4B). Thus, the collection of T-cell epitopes in the MNP provided enhanced protection against the pandemic strain of IAV.

To understand the immunological basis for the protection afforded by the PR8 NP and MNP and the differences in the degree of protection induced by MNP and PR8 NP vaccinations, we quantified CD8 and CD4 T-cell responses to the immunodominant epitopes NP366 and NP311, respectively. Surprisingly, despite a >90% reduction in viral load, NP366-specific CD8 T cells and NP311-specific CD4 T cells in the lungs of IAV-CA04-challenged mice showed no evidence of recall expansion (Fig. 4C), unlike the robust recall T-cell responses following challenge with PR8 and IAV-Aichi viruses (Fig. 2 and 3).

To understand the lack of recall responses to immunodominant epitopes following viral challenge, we compared amino acid sequences for the NP366 and NP311 epitopes in the PR8 NP, MNP, and the IAV-CA04 virus NP. While the NP366 epitope is identical in IAV PR8 NP and the MNP, the NP366 epitope of CA04 contained a single substitution at the sixth amino acid (methionine [M] to valine [V] substitution) (Fig. 4D), which is known to be sufficient to abrogate recognition by CD8 T cells that are specific to the D^b^/NP366 complex (22). After the challenge of PR8 or MNP-vaccinated mice with IAV-CA04, neither the PR8- and MNP-derived NP366 peptides nor the CA04-derived NP366 peptide induced cytokine expression in lung CD8 T cells (Fig. 4E). Moreover, 8 days post-MNP vaccination, lung T cells showed minimal cytokine production by CD8 T cells upon stimulation with the NP366-CA04 peptide (Fig. 4F). Thus, the lack of recall expansion of vaccine-induced memory NP366-specific CD8 T cells can be explained by the loss of their reactivity to the NP366 epitope of the IAV-CA04 virus. For the MHC II-restricted NP 311–325 epitope, the tyrosine (Y; at the third position) and isoleucine (I; at the sixth position) were substituted with valine (V) and methionine (M), respectively (Fig. 4D). Like what was observed for CD8 T cells, NP311-specific CD4 T cells induced by the PR8 or MNP vaccine did not exhibit cross-reactivity to stimulation with the NP311-CA04 peptide (Fig. 4E and F). Thus, the amino acid substitutions at the third and sixth residues of the NP311 epitope in the CA04 NP were sufficient to abrogate recognition by vaccine-elicited NP311-specific memory CD4 T cells. In summary, amino acid substitutions in the immunodominant epitopes of the CA04 NP likely abolished the ability of pre-existing vaccine-induced immunodominant epitope-specific memory T cells to recognize and respond to CA04 challenge.

### Broader reactivity of CD4 T cells elicited by mosaic NP is associated with enhanced control of pandemic influenza virus

**Fig 5:**
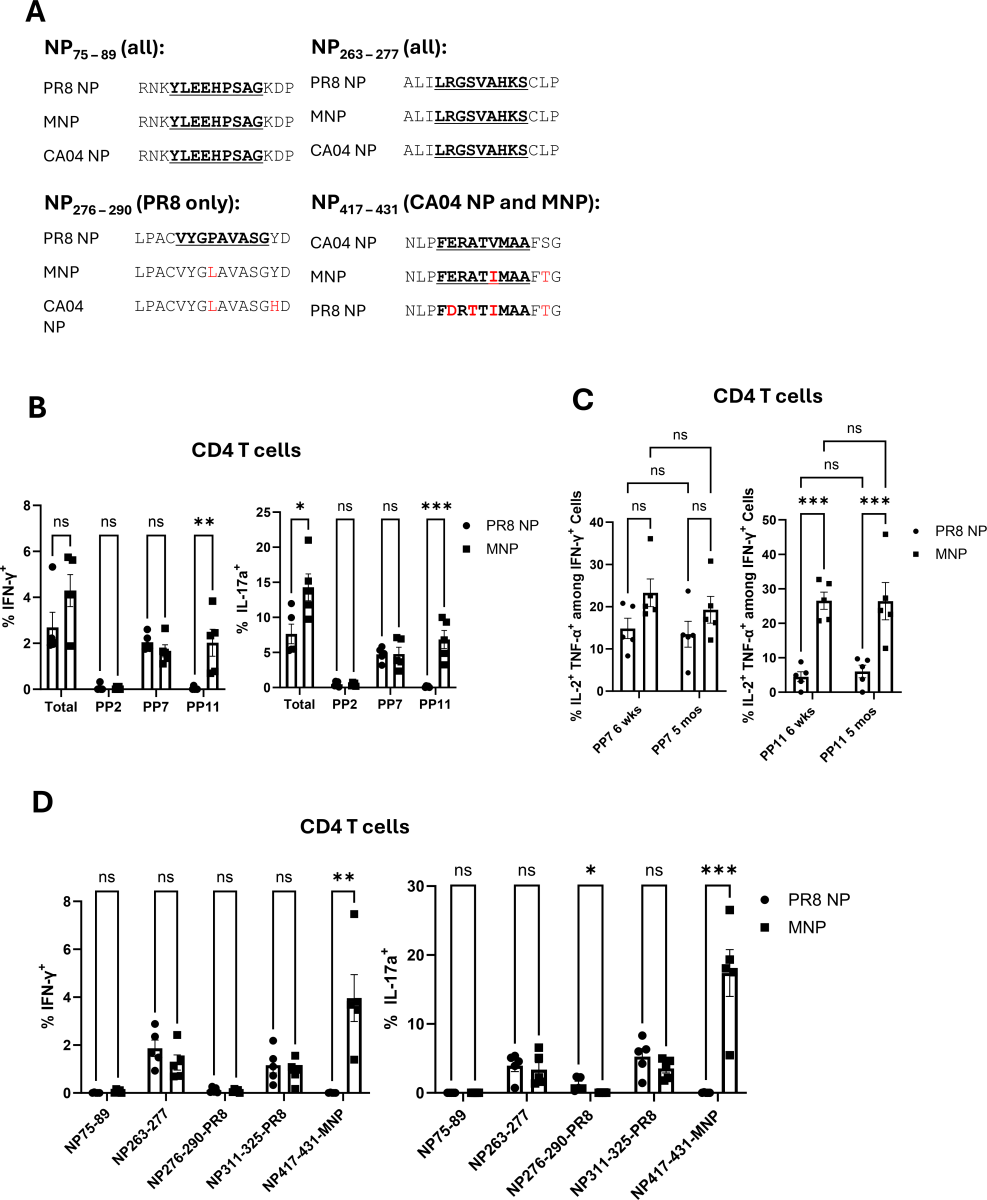
Quantification of epitope-specific CD4 T cells in mice vaccinated with PR8 NP or MNP. Peptide-specific cytokine-producing T cells were quantified by *ex vivo* stimulation with the peptides after vaccination with PR8-NP or MNP (8 days after booster vaccination), or at 6 days after challenge of vaccinated mice with the H1N1 influenza A/California/04/2009 strain. (**A**) Highly ranked epitope sequences selected through epitope prediction were compared among PR8 NP, MNP, and CA04 NP. (**B**) Cytokine-producing CD4 T cells were quantified on the 6th day after challenge with IAV-CA04 by *ex vivo* stimulation with peptide pools derived from the NP protein of H1N1 A/California/04/2009 pdm09 strain. The “Total” represents the sum of the frequency values of all 12 peptide pools that are above 0, after subtracting the value of the media control. (**C**) Polyfunctional CD4 T cells responsive to peptide pools were quantified in mice challenged with IAV-CA04 at 6 weeks or 5 months after booster vaccination. (**D**) Cytokine-producing CD4 T cells were quantified by *ex vivo* stimulation with synthetic peptides predicted by MHC Class II epitope prediction tools on the 8th day after booster vaccination. Planned pairwise group comparisons were conducted using a two-way ordinary ANOVA followed by Fisher’s least-significant difference (LSD) test, or an unpaired t test for two-group comparisons. *, **, ***, and **** denote significance at *P* < 0.05, 0.01, 0.001, and 0.0001, respectively. "ns" indicates results that are “not significant.” The data in each graph represent the mean ± SEM.

Because vaccine-induced immunity to IAV-CA04 cannot be explained by recall responses to immunodominant epitopes NP366 and NP311, we hypothesized that T-cell responses to other epitopes in the MNP mediate viral control in the lungs. Therefore, we conducted epitope prediction restricted to mouse MHC Class I and II for CA04, PR8, and MNP and compared the predicted amino acid sequences among the three proteins. The top 10 predicted regions are summarized in Tables 2 and 3. The CD8 T-cell epitope prediction identified that the top six epitope sequences covered the same region across CA04 NP, PR8 NP, and MNP. However, as observed for NP366 in CA04 NP, not all the predicted epitope sequences were identical across NP proteins. It is noteworthy that, the MNP design incorporated the maximum number of possible MHC I-restricted 9-mer epitopes. Here, we assessed whether such a 9-mer-based design altered MHC II-restricted CD4 T-cell epitopes in the MNP. As expected, the historical epitope in the NP311 region was predicted for PR8 NP and MNP, but not for the CA04 NP. However, highly ranked epitopes were predicted in the three regions spanning amino acids 417–431, 263–277, and 75–89 of the CA04 NP, and among the three, regions 263–277 and 75–89 were also predicted for PR8 NP and MNP (Fig. 5A). Interestingly, the 417–431 region was predicted for MNP and CA04 NP, but not PR8 NP. Next, to validate the *in-silico* epitope prediction, we examined the antigenic reactivity of lung T cells from vaccinated mice challenged with IAV-CA04 by stimulating with IAV-CA04 NP peptide pools composed of 14–16 amino acids or synthetic peptides derived from the top 10 predicted CD8 T-cell epitopes listed in Table 2 (Fig. S2). None of the peptides stimulated CD8 T cells to produce IFN-γ or IL-17a at levels above the media control, indicating a lack of notable recall expansion. As a surrogate for CD8 T-cell activation in the lungs of virally challenged mice, we measured granzyme B levels in activated CD44^HI^ CD8 T cells, and PMAI-induced IFN-γ/IL-17a production. The percentages of granzyme B^+ve^, IFN-γ-producing, or IL-17a-producing CD8 T cells were not significantly different in the lungs of PR8 NP- and MNP-vaccinated mice (Fig. S3). Thus, differences in CD8 T-cell recall responses did not account for improved IAV-CA04 viral control in the lungs of MNP-vaccinated mice. In contrast to CD8 T cells, we readily detected cytokine-producing CD4 T cells reactive to peptide pool (PP) 7 and PP11, covering the regions 263–277 and 417–431, respectively (Fig. 5B; Fig. S2A). No response was observed in either group for PP2 that corresponded to the predicted 75–89 region. It is noteworthy that both PR8 NP and MNP elicited comparable responses to PP7, but only MNP-induced CD4 T cells that were reactive to PP11. Unlike the response to the vaccine-specific NP311 epitope following the IAV-CA04 challenge, the proportion of polyfunctional (multi-cytokine-producing) CD4 T cells cross-reactive to CA04 NP remained stable even 5 months post-vaccination (Fig. 5C).

**TABLE 2:** Top 10 predicted MHC Class I epitope sequences from the nucleoprotein that are restricted to a specific H-2 allele in C57BL/6 mice

Allele	Start	End	Peptide	Score	% Rank
CA04 NP					
H-2-Db	366	374	ASNENVETM^*a*^	0.9906	0.01
H-2-Db	55	63	RLIQNSITI	0.8747	0.01
H-2-Kb	299	307	VGIDPFKLL	0.7165	0.04
H-2-Kb	146	154	ATYQRTRAL	0.7154	0.04
H-2-Db	296	304	YSLVGIDPF	0.7148	0.03
H-2-Kb	217	225	VAYERMCNI	0.6726	0.05
H-2-Db	305	313	KLLQNSQVV	0.5507	0.07
H-2-Db	373	381	TMDSNTLEL	0.5092	0.08
H-2-Kb	135	143	HIMIWHSNL	0.4217	0.18
H-2-Kb	344	352	SSFIRGKKV	0.3023	0.29
Mosaic NP					
H-2-Db	366	374	ASNENMETM^*a*^	0.9885	0.01
H-2-Db	55	63	RLIQNSITI	0.8747	0.01
H-2-Kb	299	307	VGIDPFRLL	0.7754	0.03
H-2-Kb	146	154	ATYQRTRAL	0.7153	0.04
H-2-Db	296	304	YSLVGIDPF	0.7148	0.03
H-2-Kb	217	225	IAYERMCNI	0.6309	0.06
H-2-Kb	344	352	SSFIRGTRV	0.6273	0.06
H-2-Db	181	189	AAVKGVGTM	0.4392	0.1
H-2-Db	363	371	VQIASNENM	0.3804	0.13
H-2-Kb	256	264	LIFLARSAL	0.2318	0.39
PR8 NP					
H-2-Db	366	374	ASNENMETM^*a*^	0.9885	0.01
H-2-Db	55	63	RLIQNSLTI	0.8608	0.02
H-2-Kb	299	307	VGIDPFRLL	0.7754	0.03
H-2-Kb	146	154	ATYQRTRAL	0.7154	0.04
H-2-Db	296	304	YSLVGIDPF	0.7148	0.03
H-2-Kb	217	225	IAYERMCNI	0.6309	0.06
H-2-Db	181	189	AAVKGVGTM	0.4392	0.1
H-2-Db	336	344	AAFEDLRVL	0.4205	0.11
H-2-Db	363	371	VQIASNENM	0.3804	0.13
H-2-Kb	135	143	HMMIWHSNL	0.3362	0.24

^*a*^ Predicted peptide sequence that covers PR8 NP_366-374_ region.

**TABLE 3:** Top 10 predicted MHC Class II epitope sequences from the nucleoprotein that are restricted to a specific H-2 allele in C57BL/6 mice

Allele	Start	End	Core peptide	Peptide	Score	% Rank
CA04 NP						
H2-IAb	417	431	FERATVMAA	NLPFERATVMAAFSG	0.5129	1.6
H2-IAb	416	430	FERATVMAA	RNLPFERATVMAAFS	0.4371	2.2
H2-IAb	263	277	LRGSVAHKS	ALILRGSVAHKSCLP	0.3883	2.6
H2-IAb	75	89	YLEEHPSAG	RNKYLEEHPSAGKDP	0.3404	3.1
H2-IAb	77	91	EEHPSAGKD	KYLEEHPSAGKDPKK	0.3438	3.1
H2-IAb	76	90	EEHPSAGKD	NKYLEEHPSAGKDPK	0.3259	3.3
H2-IAb	415	429	FERATVMAA	QRNLPFERATVMAAF	0.3197	3.4
H2-IAb	261	275	LRGSVAHKS	RSALILRGSVAHKSC	0.2961	3.7
H2-IAb	262	276	LRGSVAHKS	SALILRGSVAHKSCL	0.3003	3.7
H2-IAb	260	274	LRGSVAHKS	ARSALILRGSVAHKS	0.2589	4.3
Mosaic NP						
H2-IAb	310	324	YSLIRPNEN^*a*^	SQVYSLIRPNENPAH	0.4545	2
H2-IAb	309	323	YSLIRPNEN^*a*^	NSQVYSLIRPNENPA	0.403	2.5
H2-IAb	263	277	LRGSVAHKS	ALILRGSVAHKSCLP	0.3883	2.6
H2-IAb	75	89	YLEEHPSAG	RNKYLEEHPSAGKDP	0.3404	3.1
H2-IAb	308	322	YSLIRPNEN^*a*^	QNSQVYSLIRPNENP	0.341	3.1
H2-IAb	311	325	YSLIRPNEN^*a*^	QVYSLIRPNENPAHK	0.3436	3.1
H2-IAb	77	91	EEHPSAGKD	KYLEEHPSAGKDPKK	0.3438	3.1
H2-IAb	76	90	EEHPSAGKD	NKYLEEHPSAGKDPK	0.3259	3.3
H2-IAb	261	275	LRGSVAHKS	RSALILRGSVAHKSC	0.2961	3.7
H2-IAb	417	431	FERATIMAA	NLPFERATIMAAFTG	0.297	3.7
PR8 NP						
H2-IAb	276	290	VYGPAVASG	LPACVYGPAVASGYD	0.8209	0.3
H2-IAb	277	291	VYGPAVASG	PACVYGPAVASGYDF	0.795	0.35
H2-IAb	275	289	VYGPAVASG	CLPACVYGPAVASGY	0.6831	0.75
H2-IAb	278	292	VYGPAVASG	ACVYGPAVASGYDFE	0.5242	1.5
H2-IAb	310	324	YSLIRPNEN^*a*^	SQVYSLIRPNENPAH	0.4545	2
H2-IAb	274	288	VYGPAVASG	SCLPACVYGPAVASG	0.4079	2.4
H2-IAb	309	323	YSLIRPNEN^*a*^	NSQVYSLIRPNENPA	0.403	2.5
H2-IAb	263	277	LRGSVAHKS	ALILRGSVAHKSCLP	0.3883	2.6
H2-IAb	75	89	YLEEHPSAG	RNKYLEEHPSAGKDP	0.3404	3.1
H2-IAb	308	322	YSLIRPNEN^*a*^	QNSQVYSLIRPNENP	0.341	3.1

^*a*^ Predicted core peptide sequence that covers PR8 NP_311-325_ region.

Next, we determined whether vaccination alone with PR8 NP or MNP elicited PP7 and PP11 reactive CD4 T cells, at 8 days following boost. Data in Fig. S4 show that vaccination alone elicited CD4 T cells specific to NP311, PP7, and/or PP11. As observed in recall responses (Fig. 5B), CD4 T cells responsive to PP11 were only elicited by MNP. For rigor, we compared the reactivity to the *in silico* predicted CD8 and CD4 T-cell epitopes in mice vaccinated with PR8 NP or MNP to assess the breadth of the vaccine-induced T-cell response. For CD8 T cells, none of the 14 tested 9-mer peptides demonstrated any detectable reactivity (Fig. S5). Consequently, the historic NP366 epitope appears to be the sole predominant CD8 T-cell epitope in C57BL/6 mice. On the other hand, CD4 T-cell reactivity to single peptides corresponding to each peptide pool was consistent with the results from peptide pool stimulation (Fig. 5D), confirming that the 417–431 epitope from CA04 and MNP is cross-reactive. Taken together, these data suggested that MNP elicited a broader range of NP-specific CD4 T cells than the PR8 NP, which might explain better protection against IAV-CA04.

## DISCUSSION

Influenza virus pandemics have had indelible effects on human civilization, and a major public health goal has been to develop a universal influenza vaccine that can provide broad protection against contemporary and pandemic strains of IAV (23, 24). Toward this goal, there is ongoing significant effort in developing strategies for eliciting antibody responses to the highly conserved region of IAV hemagglutinin (24, 25). Likewise, there is impetus to develop T-cell-based vaccines targeting the less mutable and conserved internal viral proteins, such as the NP (3, 26). In this study, we designed an MNP vaccine targeting conserved epitopes within the influenza IAV NP, to induce broad and robust anti-influenza T-cell responses, providing potential protection against a variety of influenza strains. IAV NP was selected for mosaic design due to its well-characterized immunodominant T-cell epitopes in C57BL/6 mice and its critical, highly conserved role in the viral life cycle. While other internal antigens, such as M1 and PB1, also display conservation, they may exhibit more host-specific variability relative to NP (27). NP is abundant in infected cells and less susceptible to the extensive antigenic drift observed in HA (28), making it a suitable candidate for broad T-cell targeting. However, mosaic vaccine strategies have been successfully applied to HA to elicit broadly reactive antibodies against diverse influenza strains (18–20). Incorporating multiple mosaic antigens from several viral proteins could further enhance the breadth of immunity, potentially conferring improved and durable protection against a wide spectrum of influenza variants.

Here we report that the MNP vaccine elicited diverse responses to immunodominant and subdominant epitopes, leading to the substantial reduction in lung viral load upon challenge with both seasonal H1N1 and H3N2 as well as the pandemic H1N1 strain, indicating its effectiveness across different influenza subtypes. These results have implications in utilizing mosaics of internal viral proteins of IAV as candidate antigens for diversifying T-cell responses, in T-cell-based universal influenza vaccines.

What is the advantage of a mosaic protein vaccine versus a consensus sequence-based vaccine? While consensus sequence-based vaccines directly address the problem of pathogen diversity and have shown success in eliciting immunity to influenza virus (29–31), averaging sequences can result in a bias toward input sequences without specific consideration to maximize immune coverage of diverse epitopes (32). In the current study, the MNP was constructed from a compilation of epitopes derived from over 7422 NP sequences across human, swine, and avian influenza strains, ensuring comprehensive epitope representation. This approach allowed the MNP to exhibit significantly higher coverage of 9-mer epitope sequences derived from all NPs compared to the single-strain derived NP.

The central role of memory T-cell responses in the context of a T-cell epitope-based mosaic vaccine against influenza is underscored by their ability to rapidly recall and execute immune functions upon pathogen re-exposure. Memory CD4 T cells, in particular, have been shown to significantly impact heterosubtypic immunity against various influenza strains, enhancing both the speed and efficacy of viral clearance (33). These cells not only aid in the immediate control of the virus through cytokine production but also bolster the activation of memory B cells and cytotoxic T cells, critical for long-term immunity and cross-protection against divergent influenza subtypes. It is important to note that vaccination of human and animal populations would take place in the immune context of pre-existing CD4 and CD8 T-cell immunity to influenza viruses. The phenomenon of original antigenic sin, where prior exposure to influenza strains can influence the response to new vaccines, highlights the complex interplay of pre-existing immunity in vaccine effectiveness (34). Pre-existing memory CD4+ T cells, shaped by earlier infections, can either potentiate or hinder the immune response to newly encountered influenza viruses depending on the similarity between the encountered antigens (35). This aspect of memory T-cell biology is key in understanding the varying outcomes of MNP vaccine deployment in diverse populations, which may differ significantly in their historical exposure to influenza. Optimizing vaccine designs to account for these immunological landscapes, by targeting broad and cross-reactive T-cell epitopes, could enhance vaccine efficacy universally, circumventing the limitations imposed by original antigenic sin and improving global preparedness against pandemic influenza threats.

It is noteworthy that the MNP elicited responses to both well-characterized immunodominant and *in silico*-predicted subdominant T-cell epitopes. Challenges with IAV strains that differed in terms of the conservation of immunodominant class I and class II-restricted epitopes (NP366 and NP311) provided new insights into the mechanisms of protective immunity. It was evident that matches in NP366 and/or NP311 epitopes between the MNP and the viral NP led to robust protection, that transcended viral subtypes, that is, H1N1 and H3N2 viruses. Interestingly, MNP provided significantly greater protection against the pandemic CA04/H1N1 challenge, as compared to PR8 NP. Since CA04 failed to elicit recall T-cell responses to the immunodominant epitopes NP366 or NP311 in the MNP or PR8 NP, enhanced protection in MNP-vaccinated mice was instead associated with distinct CD4 T-cell responses to an epitope contained in the 417–431 regions, elicited only by the MNP. Depletion of CD8 and/or CD4 T cells will clarify the individual role of T-cell subsets in protection. These findings underscore that MNP vaccine-induced heterosubtypic T-cell immunity targeting conserved subdominant epitopes could prove effective even in instances where immunodominant epitopes are absent in the pandemic viral strain. In addition, it is worth noting that our experiments were conducted on inbred strains of C57BL/6 mice, and it is possible that vaccinated humans, poultry, or swine will recognize a broader array of T-cell epitopes within the MNP, as the sequence was generated from viruses isolated from these species. While the mosaic vaccine algorithm does not specifically account for host HLA genetics, and instead functions to identify a sequence that maximizes 9-mer coverage against all input sequences, experiments in target species would be needed to determine if vaccination with the MNP results in a greater breadth of T-cell responses as compared to vaccination with natural sequences.

In this study, we formulated MNP in a combination adjuvant consisting of a carbomer-based nano-emulsion (Adjuplex) and GLA (TLR-4 agonist) and administered the vaccine intranasally. Our previous research has demonstrated the effectiveness of this combination adjuvant in harnessing Adjuplex’s capacity to engage the cross-presentation pathway of antigen presentation while utilizing the immunomodulatory properties of GLA (21, 36, 37). This particular synergy stimulates CD4 and CD8 T cells that produce both IFN-γ and IL-17 and induces exceptionally strong tissue-resident memory CD8 and CD4 T-cell responses within the airways and lungs, resulting in durable protection against both IAV and SARS-CoV-2 (21, 37, 38). Moreover, we found that T-cell-based vaccines formulated with Adjuplex confer protective immunity against IAV exclusively through intranasal administration, implicating tissue-resident memory T cells in this process (39). Therefore, the selection of adjuvant and vaccination routes not only influences the localization of memory T cells (systemic/vascular versus tissue resident in the lungs and airways) and protective immunity but also might impact the diversity of T-cell responses. Hence, the careful selection of adjuvants and vaccination routes is critical for the development and evaluation of T-cell-based vaccines.

In addition to classical IFN-γ-dominated responses, recent work—including our own—has highlighted the contribution of IL-17-producing T cells to protective immunity against influenza. Our previous investigations have demonstrated that IL-17-expressing CD4^+^ and CD8^+^ T cells correlate with improved viral clearance (21, 37). Notably, T cells co-expressing both IFN-γ and IL-17 exhibited even stronger correlations with protection, suggesting that a multifaceted cytokine profile may enhance antiviral defenses. These findings align with other studies indicating that bona fide Th17 cells, which are generated during influenza infection with and without Th1 functional plasticity, support the host response and can broaden the protective T-cell repertoire (40, 41).

In this manuscript, we present, for the first time, evidence that MNP formulated in a nano-emulsion-based combination adjuvant is immunogenic, triggering a diverse T-cell response and offering effective lung immunity against both seasonal and pandemic IAVs in mice. Consequently, a mosaic of NP containing T-cell epitopes sourced from a variety of avian, swine, and human IAVs could serve as a broadly protective antigen, guarding humans against a wide range of IAV strains.

## MATERIALS AND METHODS

### Experimental animals

Seven- to 12-week-old C57BL/6J mice were purchased from restricted-access specific-pathogen-free (SPF) mouse breeding colonies at the University of Wisconsin–Madison Breeding Core Facility or from Jackson Laboratory. All mice were housed in SPF conditions in the animal facilities at the University of Wisconsin-Madison.

### Construction of mosaic NP

The available human, avian, and swine influenza NP sequences were downloaded from the Influenza Research Database (fludb.org/Genbank, accessed October 2018). Subsequently, any duplicate, incomplete, or highly dissimilar sequences were pruned. The resulting 7422 NP sequences were subjected to an alignment that was generated using Unipro UGENE software (42), and applying the MUltiple Sequence Comparison by Log- Expectation (MUSCLE) algorithm. This sequence alignment was used to generate a 9-mer mosaic protein (www.hiv.lanl.gov), using their mosaic design tool. Briefly, the alignment sequences were uploaded to the mosaic vaccine design tool, and parameters were selected to specify the output of one mosaic sequence, including a population recombination size of 500 and a rare threshold of 3. The generated mosaic sequence was analyzed for epitope coverage using tools available at hiv.lanl.gov/content/sequence/MOSAIC/. In essence, the MNP incorporates into the sequence, a maximum number of potential 9-mer CD8 T-cell epitopes and also preserves the overall structure and function of the NP. The MNP was synthesized utilizing the *E. coli* BL21 (DE3) system and subsequently purified using Ni-NTA affinity chromatography. The protein, which exhibited an endotoxin level of <0.1 EU/mL, was employed as an antigen in the vaccine formulation.

### Vaccines and vaccination

PR8 NP was purchased from Sino Biological Inc (Beijing, China), and the synthetic MNP was acquired from Creative BioMart (NY, USA). The synthetic monophosphoryl lipid A adjuvant, Glucopyranosyl Lipid Adjuvant (GLA) was purchased from Avanti Polar Lipids, Inc. (Alabaster, AL). Adjuplex is a proprietary preparation consisting of an emulsion of polyacrylic acid and soy lecithin, provided by Advanced BioAdjuvants, LLC. All vaccinations were administered intranasally to anesthetized mice in 50 µL saline with 10 µg PR8 NP or MNP with the adjuvant: 5% Adjuplex (ADJ) +5 µg GLA (ADJ + GLA). For all studies, mice were vaccinated twice at an interval of 3 weeks (21).

### Viral challenge and viral titration

Influenza virus strain A/PR/8/34 H1N1 (IAV-PR8) was provided by Dr. Robert Webster (St. Jude’s Children’s Hospital). Influenza virus strain A/Aichi/2/1968 H3N2 (IAV-Aichi) and strain A/California/04/2009 H1N1 (IAV-CA04) were grown in the laboratory of Dr. Yoshihiro Kawaoka (43, 44). For viral challenge studies, mice were inoculated intranasally with 500 plaque-forming units (PFU) of IAV-PR8 or 1 × 10^5^ PFU of IAV-Aichi or 5,000 PFU of IAV-CA04 strains of influenza A virus in 50 µL PBS. Lung tissues (left lobe) were collected on the 6th day after the viral challenge for the viral titration by a plaque assay. Briefly, lung tissues were homogenized using a tissue homogenizer and then centrifuged for 3 minutes at 2,000 RPM in a microcentrifuge. Supernatants were subsequently collected, and 10-fold serial dilutions of these supernatants were utilized for titration purposes. Madin-Darby canine kidney (MDCK) cells were obtained from ATCC (ATCC; Manassas, VA, USA) and propagated in growth media containing Modified Eagle’s Medium (MEM) with 10% fetal bovine serum (FBS; Hyclone, Logan, UT), 2 mM L-glutamine, 1.5 g/L sodium bicarbonate, non-essential amino acids, 100 U/mL of penicillin, 100 µg/mL of streptomycin, and incubated at 37°C in 5% CO_2_. MDCK cells grown to 90% confluency were infected with 100 µL of serial dilutions of samples and incubated for 1 hour at 37°C. Cells were then washed with PBS and incubated in media containing 1% SeaPlaque agarose (Lonza, Basel, Switzerland) and 10 µg/mL of TPCK-treated trypsin. After 48 hours of incubation period, cells were fixed in 10% neutral buffered formalin (NBF), agarose plugs were then removed, and distinct plaques were counted at specific dilutions to determine the PFU of the virus per sample.

### Flow cytometry

Single-cell suspensions from the lung tissues were prepared using standard techniques as previously described (21). Prior to surface staining, cells were stained for viability with Ghost Dye 780 (Tonbo Biosciences, San Diego, CA) according to the manufacturer’s instructions. Following this, the I-A^b^/NP311 tetramer (diluted 1:150 in RPMI1640 containing 10% FBS) was incubated at 37°C for 90 minutes. Subsequently, the D^b^/NP366 tetramer (diluted 1:150 in brilliant staining buffer (BSB, BD Biosciences) was incubated at 4°C for 60 minutes along with other fluorochrome-labeled antibodies targeting surface antigens, followed by fixation with 2% paraformaldehyde. All samples were acquired on LSRFortessa (BD Biosciences) and analyzed with FlowJo V.10 software (TreeStar, Ashland, OR).

For intracellular cytokine staining, cells were stimulated for 5 hours at 37°C in the presence of brefeldin A (1 µL/mL, GolgiPlug, BD Biosciences) and human recombinant IL-2 (10 U/well), along with the following peptides: PR8 NP366 and PR8 NP311 peptides were synthesized by Thinkpeptides, ProImmune Ltd., Oxford, UK. Peptides from the top 10 epitope prediction lists including Aichi NP366 and CA04 NP366 were synthesized by GenScript, NJ, USA (listed in Table S1). We created 12 peptide pools (PPs; 10 peptides/pool) from a total of 122-peptide array (NR-18976, BEI Resources), which spans the nucleocapsid protein of the A/California/04/2009 (H1N1) pdm09 strain of influenza virus; peptides are 14 to 16-mers, with 10–12 amino acid overlaps (Table S1). Following stimulation, cells were transferred to a 96-well round-bottom plate (Corning) and stained for viability using the same dye as above. Subsequently, cells were fixed and permeabilized using the Cytofix/Cytoperm kit (BD Biosciences, Franklin Lakes, NJ). Samples were then stained with antibodies specific to the cytokines listed in Table S1 in perm wash buffer. All staining procedures were conducted on ice. Peptide-specific responses were calculated by subtracting the cytokine expression values obtained from the media controls from those obtained after peptide stimulation for each sample, and negative values were replaced with zero.

### Epitope prediction

The sequences of potential 9-mer CD8 T-cell epitopes restricted by H2-D^b^ and H2-K^b^ from CA04 NP, PR8 NP, and MNP were predicted using NetMHCpan-4.1 (http://tools.iedb.org/mhci/) (45). In addition, potential 15-mer CD4 T-cell epitope sequences restricted by H2-IA^b^ from the same antigens were predicted using NetMHCIIpan-4.1 (http://tools.iedb.org/mhcii/) (45, 46).

### Network graph construction

The 7422 Influenza A virus sequences included in the MNP were analyzed by extracting all unique 9-mer subsequences using custom R scripts developed to parse amino acid sequences and generate overlapping 9-mer windows. Pairwise Jaccard similarity coefficients were calculated to assess the proportion of shared 9-mers between all pairs of sequences. Hierarchical clustering was performed on the resulting similarity matrix using the dendextend package in R to group highly similar sequences into distinct clusters. From each cluster, one representative sequence was selected. A network graph was constructed where nodes represent these representative sequences and edges indicate a shared 9-mer overlap of Jaccard similarity ≥0.4, utilizing the igraph package. Node attributes were assigned based on host origin. The network was visualized with the ggraph package in R, employing a force-directed layout to illustrate the relationships among sequences. The code and analysis pipeline used in this study is available at DOI: 10.5281/zenodo.14398864.

### Statistical analyses

Statistical analyses were performed using GraphPad Prism 10.0 (GraphPad Software, La Jolla, CA). Planned pairwise group comparisons were conducted using a one-way ordinary ANOVA followed by Fisher’s least-significant difference (LSD) test, provided variance homogeneity was confirmed by Brown-Forsythe and Bartlett’s tests. In cases where variances were not equal, multiple comparisons were performed using Welch’s ANOVA followed by Welch’s t-tests. Two-group comparisons were made using unpaired t-tests. Significance is represented as *, **, ***, and **** for *P* < 0.05, 0.01, 0.001, and 0.0001, respectively. Viral titers were log-transformed prior to analysis, and all data are presented as mean ± SEM.

## Data Availability

The data that support the findings of this study are available from the corresponding author, M. Suresh, upon request.

## SUPPLEMENTAL MATERIAL

Supplemental figures jvi.00867-24-s0001.pdf Figures S1 to S5.

Supplemental legends jvi.00867-24-s0002.docx Legends for Fig. S1 to S5.

Table S1 jvi.00867-24-s0003.docx Antibodies and peptides.

ASM does not own the copyrights to Supplemental Material that may be linked to, or accessed through, an article. The authors have granted ASM a non-exclusive, world-wide license to publish the Supplemental Material files. Please contact the corresponding author directly for reuse.

## References

[1] Petrova VN, Russell CA. 2018. The evolution of seasonal influenza viruses. Nat Rev Microbiol 16:47–60. doi:10.1038/nrmicro.2017.118 29109554

[2] Smith DJ, Lapedes AS, de Jong JC, Bestebroer TM, Rimmelzwaan GF, Osterhaus ADME, Fouchier RAM. 2004. Mapping the antigenic and genetic evolution of influenza virus. Science 305:371–376. doi:10.1126/science.1097211 15218094

[3] Mosmann TR, McMichael AJ, LeVert A, McCauley JW, Almond JW. 2024. Opportunities and challenges for T cell-based influenza vaccines. Nat Rev Immunol 24:736–752. doi:10.1038/s41577-024-01030-8 38698082

[4] Krammer F, García-Sastre A, Palese P. 2018. Is it possible to develop a “universal” influenza virus vaccine? Potential target antigens and critical aspects for a universal influenza vaccine. Cold Spring Harb Perspect Biol 10:a028845. doi:10.1101/cshperspect.a028845 28663209 PMC6028071

[5] Isakova-Sivak I, Rudenko L. 2022. The future of haemagglutinin stalk-based universal influenza vaccines. Lancet Infect Dis 22:926–928. doi:10.1016/S1473-3099(22)00056-1 35461523

[6] Nachbagauer R, Palese P. 2020. Is a universal influenza virus vaccine possible? Annu Rev Med 71:315–327. doi:10.1146/annurev-med-120617-041310 31600454

[7] Clemens EB, van de Sandt C, Wong SS, Wakim LM, Valkenburg SA. 2018. Harnessing the power of T cells: the promising hope for a universal influenza vaccine. Vaccines (Basel) 6:18. doi:10.3390/vaccines6020018 29587436 PMC6027237

[8] Quiñones-Parra S, Grant E, Loh L, Nguyen THO, Campbell K-A, Tong SYC, Miller A, Doherty PC, Vijaykrishna D, Rossjohn J, Gras S, Kedzierska K. 2014. Preexisting CD8^+^ T-cell immunity to the H7N9 influenza A virus varies across ethnicities. Proc Natl Acad Sci U S A 111:1049–1054. doi:10.1073/pnas.1322229111 24395804 PMC3903243

[9] Sridhar S, Begom S, Bermingham A, Hoschler K, Adamson W, Carman W, Bean T, Barclay W, Deeks JJ, Lalvani A. 2013. Cellular immune correlates of protection against symptomatic pandemic influenza. Nat Med 19:1305–1312. doi:10.1038/nm.3350 24056771

[10] Wang Z, Wan Y, Qiu C, Quiñones-Parra S, Zhu Z, Loh L, Tian D, Ren Y, Hu Y, Zhang X, Thomas PG, Inouye M, Doherty PC, Kedzierska K, Xu J. 2015. Recovery from severe H7N9 disease is associated with diverse response mechanisms dominated by CD8^+^ T cells. Nat Commun 6:6833. doi:10.1038/ncomms7833 25967273 PMC4479016

[11] Wilkinson TM, Li CKF, Chui CSC, Huang AKY, Perkins M, Liebner JC, Lambkin-Williams R, Gilbert A, Oxford J, Nicholas B, Staples KJ, Dong T, Douek DC, McMichael AJ, Xu X-N. 2012. Preexisting influenza-specific CD4^+^ T cells correlate with disease protection against influenza challenge in humans. Nat Med 18:274–280. doi:10.1038/nm.2612 22286307

[12] Barouch DH, O’Brien KL, Simmons NL, King SL, Abbink P, Maxfield LF, Sun Y-H, La Porte A, Riggs AM, Lynch DM, Clark SL, Backus K, Perry JR, Seaman MS, Carville A, Mansfield KG, Szinger JJ, Fischer W, Muldoon M, Korber B. 2010. Mosaic HIV-1 vaccines expand the breadth and depth of cellular immune responses in rhesus monkeys. Nat Med 16:319–323. doi:10.1038/nm.2089 20173752 PMC2834868

[13] Santra S, Liao HX, Zhang RJ, Muldoon M, Watson S, Fischer W, Theiler J, Szinger J, Balachandran H, Buzby A, Quinn D, Parks RJ, Tsao CY, Carville A, Mansfield KG, Pavlakis GN, Felber BK, Haynes BF, Korber BT, Letvin NL. 2010. Mosaic vaccines elicit CD8^+^ T lymphocyte responses that confer enhanced immune coverage of diverse HIV strains in monkeys. Nat Med 16:324–328. doi:10.1038/nm.2108 20173754 PMC2834806

[14] Nickle DC, Jensen MA, Gottlieb GS, Shriner D, Learn GH, Rodrigo AG, Mullins JI. 2003. Consensus and ancestral state HIV vaccines. Science 299:1515–1518. doi:10.1126/science.299.5612.1515c 12624248

[15] Gaschen B, Taylor J, Yusim K, Foley B, Gao F, Lang D, Novitsky V, Haynes B, Hahn BH, Bhattacharya T, Korber B. 2002. AIDS - diversity considerations in HIV-1 vaccine selection. Science 296:2354–2360. doi:10.1126/science.1070441 12089434

[16] Fischer W, Perkins S, Theiler J, Bhattacharya T, Yusim K, Funkhouser R, Kuiken C, Haynes B, Letvin NL, Walker BD, Hahn BH, Korber BT. 2007. Polyvalent vaccines for optimal coverage of potential T-cell epitopes in global HIV-1 variants. Nat Med 13:100–106. doi:10.1038/nm1461 17187074

[17] Florek KR, Kamlangdee A, Mutschler JP, Kingstad-Bakke B, Schultz-Darken N, Broman KW, Osorio JE, Friedrich TC. 2017. A modified vaccinia Ankara vaccine vector expressing a mosaic H5 hemagglutinin reduces viral shedding in rhesus macaques. PLoS ONE 12:e0181738. doi:10.1371/journal.pone.0181738 28771513 PMC5542451

[18] Kamlangdee A, Kingstad-Bakke B, Anderson TK, Goldberg TL, Osorio JE. 2014. Broad protection against avian influenza virus by using a modified vaccinia Ankara virus expressing a mosaic hemagglutinin gene. J Virol 88:13300–13309. doi:10.1128/JVI.01532-14 25210173 PMC4249068

[19] Kamlangdee A, Kingstad-Bakke B, Osorio JE. 2016. Mosaic H5 hemagglutinin provides broad humoral and cellular immune responses against influenza viruses. J Virol 90:6771–6783. doi:10.1128/JVI.00730-16 27194759 PMC4944288

[20] Kingstad-Bakke BA, Chandrasekar SS, Phanse Y, Ross KA, Hatta M, Suresh M, Kawaoka Y, Osorio JE, Narasimhan B, Talaat AM. 2019. Effective mosaic-based nanovaccines against avian influenza in poultry. Vaccine (Auckl) 37:5051–5058. doi:10.1016/j.vaccine.2019.06.077 31300285

[21] Marinaik CB, Kingstad-Bakke B, Lee W, Hatta M, Sonsalla M, Larsen A, Neldner B, Gasper DJ, Kedl RM, Kawaoka Y, Suresh M. 2020. Programming multifaceted pulmonary T cell immunity by combination adjuvants. Cell Rep Med 1:100095. doi:10.1016/j.xcrm.2020.100095 32984856 PMC7508055

[22] Guo H, Topham DJ. 2012. Multiple distinct forms of CD8+ T cell cross-reactivity and specificities revealed after 2009 H1N1 influenza A virus infection in mice. PLoS ONE 7:e46166. doi:10.1371/journal.pone.0046166 23029425 PMC3459832

[23] Erbelding EJ, Post DJ, Stemmy EJ, Roberts PC, Augustine AD, Ferguson S, Paules CI, Graham BS, Fauci AS. 2018. A universal influenza vaccine: the strategic plan for the National Institute of Allergy and Infectious Diseases. J Infect Dis 218:347–354. doi:10.1093/infdis/jiy103 29506129 PMC6279170

[24] Harris E. 2023. NIH launches phase 1 trial of broader “universal” flu vaccine. JAMA 330:1421. doi:10.1001/jama.2023.18741 37755937

[25] Guthmiller JJ, Han J, Utset HA, Li L, Lan LY-L, Henry C, Stamper CT, McMahon M, O’Dell G, Fernández-Quintero ML, et al.. 2022. Broadly neutralizing antibodies target a haemagglutinin anchor epitope. Nature 602:314–320. doi:10.1038/s41586-021-04356-8 34942633 PMC8828479

[26] Tan MP, Tan WS, Mohamed Alitheen NB, Yap WB. 2021. M2e-based influenza vaccines with nucleoprotein: a review. Vaccines (Basel) 9:739. doi:10.3390/vaccines9070739 34358155 PMC8310010

[27] Heiny AT, Miotto O, Srinivasan KN, Khan AM, Zhang GL, Brusic V, Tan TW, August JT. 2007. Evolutionarily conserved protein sequences of influenza A viruses, avian and human, as vaccine targets. PLoS One 2:e1190. doi:10.1371/journal.pone.0001190 18030326 PMC2065905

[28] Shaw ML, Stone KL, Colangelo CM, Gulcicek EE, Palese P. 2008. Cellular proteins in influenza virus particles. PLoS Pathog 4:e1000085. doi:10.1371/journal.ppat.1000085 18535660 PMC2390764

[29] Ma N, Xia Z-W, Zhang Z-G, Nian X-X, Li X-D, Gong Z, Zhang G-M, Le Y, Zhou R, Zhang J-Y, Yang X-M. 2023. Development of an mRNA vaccine against a panel of heterologous H1N1 seasonal influenza viruses using a consensus hemagglutinin sequence. Emerg Microbes Infect 12:2202278. doi:10.1080/22221751.2023.2202278 37067355 PMC10155637

[30] Tutykhina I, Esmagambetov I, Bagaev A, Pichugin A, Lysenko A, Shcherbinin D, Sedova E, Logunov D, Shmarov M, Ataullakhanov R, Naroditsky B, Gintsburg A. 2018. Vaccination potential of B and T epitope-enriched NP and M2 against Influenza A viruses from different clades and hosts. PLoS One 13:e0191574. doi:10.1371/journal.pone.0191574 29377916 PMC5788337

[31] Zheng M, Luo J, Chen Z. 2014. Development of universal influenza vaccines based on influenza virus M and NP genes. Infection 42:251–262. doi:10.1007/s15010-013-0546-4 24178189

[32] Cohen KW, Fiore-Gartland A, Walsh SR, Yusim K, Frahm N, Elizaga ML, Maenza J, Scott H, Mayer KH, Goepfert PA, et al.. 2023. Trivalent mosaic or consensus HIV immunogens prime humoral and broader cellular immune responses in adults. J Clin Invest 133. doi:10.1172/JCI163338 PMC9927951 36787249

[33] McKinstry KK, Strutt TM, Swain SL. 2011. Hallmarks of CD4 T cell immunity against influenza. J Intern Med 269:507–518. doi:10.1111/j.1365-2796.2011.02367.x 21362069 PMC3395075

[34] Henry C, Palm A-KE, Krammer F, Wilson PC. 2018. From original antigenic sin to the universal influenza virus vaccine. Trends Immunol 39:70–79. doi:10.1016/j.it.2017.08.003 28867526 PMC5748348

[35] Zens KD, Farber DL. 2015. Memory CD4 T cells in influenza. Curr Top Microbiol Immunol 386:399–421. doi:10.1007/82_2014_401 25005927 PMC4339101

[36] Lee W, Kingstad-Bakke B, Paulson B, Larsen A, Overmyer K, Marinaik CB, Dulli K, Toy R, Vogel G, Mueller KP, Tweed K, Walsh AJ, Russell J, Saha K, Reyes L, Skala MC, Sauer JD, Shayakhmetov DM, Coon J, Roy K, Suresh M. 2021. Carbomer-based adjuvant elicits CD8 T-cell immunity by inducing a distinct metabolic state in cross-presenting dendritic cells. PLoS Pathog 17:e1009168. doi:10.1371/journal.ppat.1009168 33444400 PMC7840022

[37] Kingstad-Bakke B, Toy R, Lee W, Pradhan P, Vogel G, Marinaik CB, Larsen A, Gates D, Luu T, Pandey B, Kawaoka Y, Roy K, Suresh M. 2020. Polymeric pathogen-like particles-based combination adjuvants elicit potent mucosal T cell immunity to influenza A virus. Front Immunol 11:559382. doi:10.3389/fimmu.2020.559382 33767689 PMC7986715

[38] Kingstad-Bakke B, Lee W, Chandrasekar SS, Gasper DJ, Salas-Quinchucua C, Cleven T, Sullivan JA, Talaat A, Osorio JE, Suresh M. 2022. Vaccine-induced systemic and mucosal T cell immunity to SARS-CoV-2 viral variants. Proc Natl Acad Sci U S A 119:e2118312119. doi:10.1073/pnas.2118312119 35561224 PMC9171754

[39] Gasper DJ, Neldner B, Plisch EH, Rustom H, Carrow E, Imai H, Kawaoka Y, Suresh M. 2016. Effective respiratory CD8 T-cell immunity to influenza virus induced by intranasal carbomer-lecithin-adjuvanted non-replicating vaccines. PLoS Pathog 12:e1006064. doi:10.1371/journal.ppat.1006064 27997610 PMC5173246

[40] Dhume K, Finn CM, Devarajan P, Singh A, Tejero JD, Prokop E, Strutt TM, Sell S, Swain SL, McKinstry KK. 2022. Bona fide Th17 cells without Th1 functional plasticity protect against influenza. J Immunol 208:1998–2007. doi:10.4049/jimmunol.2100801 35338093 PMC9012674

[41] Hamada H, Garcia-Hernandez M de la L, Reome JB, Misra SK, Strutt TM, McKinstry KK, Cooper AM, Swain SL, Dutton RW. 2009. Tc17, a unique subset of CD8 T cells that can protect against lethal influenza challenge. J Immunol 182:3469–3481. doi:10.4049/jimmunol.0801814 19265125 PMC2667713

[42] Okonechnikov K, Golosova O, Fursov M, Team U. 2012. Unipro UGENE: a unified bioinformatics toolkit. Bioinformatics 28:1166–1167. doi:10.1093/bioinformatics/bts091 22368248

[43] Ito T, Gorman OT, Kawaoka Y, Bean WJ, Webster RG. 1991. Evolutionary analysis of the influenza A virus M gene with comparison of the M1 and M2 proteins. J Virol 65:5491–5498. doi:10.1128/JVI.65.10.5491-5498.1991 1895397 PMC249043

[44] Itoh Y, Shinya K, Kiso M, Watanabe T, Sakoda Y, Hatta M, Muramoto Y, Tamura D, Sakai-Tagawa Y, Noda T, et al.. 2009. In vitro and in vivo characterization of new swine-origin H1N1 influenza viruses. Nature 460:1021–1025. doi:10.1038/nature08260 19672242 PMC2748827

[45] Reynisson B, Alvarez B, Paul S, Peters B, Nielsen M. 2020. NetMHCpan-4.1 and NetMHCIIpan-4.0: improved predictions of MHC antigen presentation by concurrent motif deconvolution and integration of MS MHC eluted ligand data. Nucleic Acids Res 48:W449–W454. doi:10.1093/nar/gkaa379 32406916 PMC7319546

[46] Kaabinejadian S, Barra C, Alvarez B, Yari H, Hildebrand WH, Nielsen M. 2022. Accurate MHC motif deconvolution of immunopeptidomics data reveals a significant contribution of DRB3, 4 and 5 to the total DR immunopeptidome. Front Immunol 13:835454. doi:10.3389/fimmu.2022.835454 35154160 PMC8826445

