# Supplementary material for: Diversifying T-cell responses: safeguarding against pandemic influenza with mosaic nucleoprotein
Source: J Virol. 2025 Feb 3;99(3):e00867-24. doi: 10.1128/jvi.00867-24 (PMC11915837; doi:10.1128/jvi.00867-24)

Diversifying T Cell Responses: Safeguarding Against Pandemic Influenza with Mosaic Nucleoprotein

Supplemental Figures

Supplemental Figure 1.

A

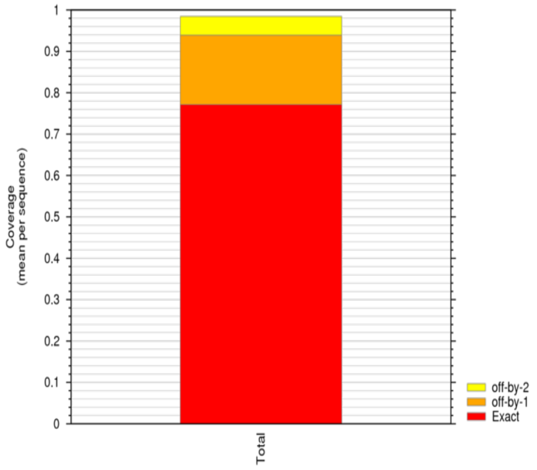

B

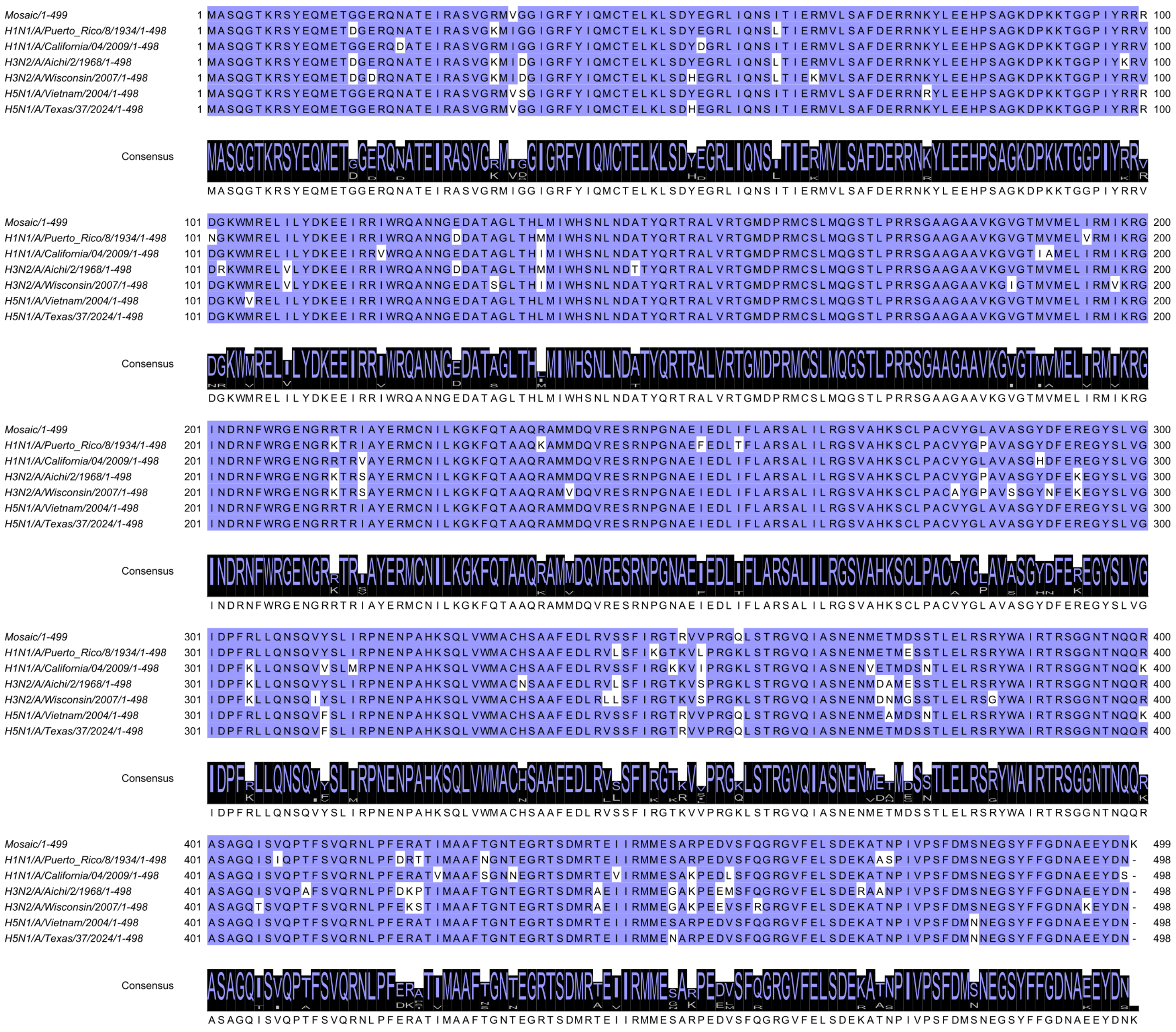

Supplemental Figure 2.

A

CD4 T cells

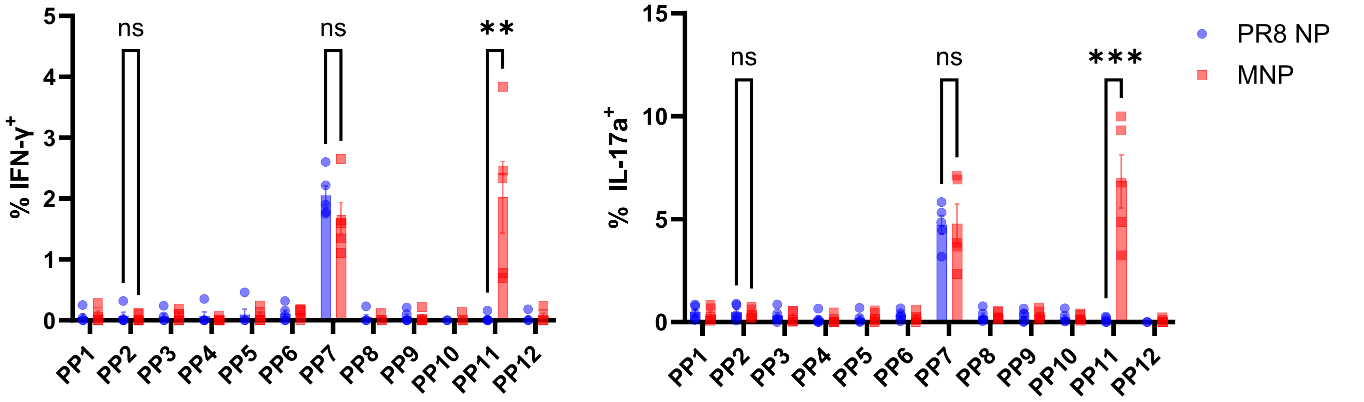

CD8 T cells

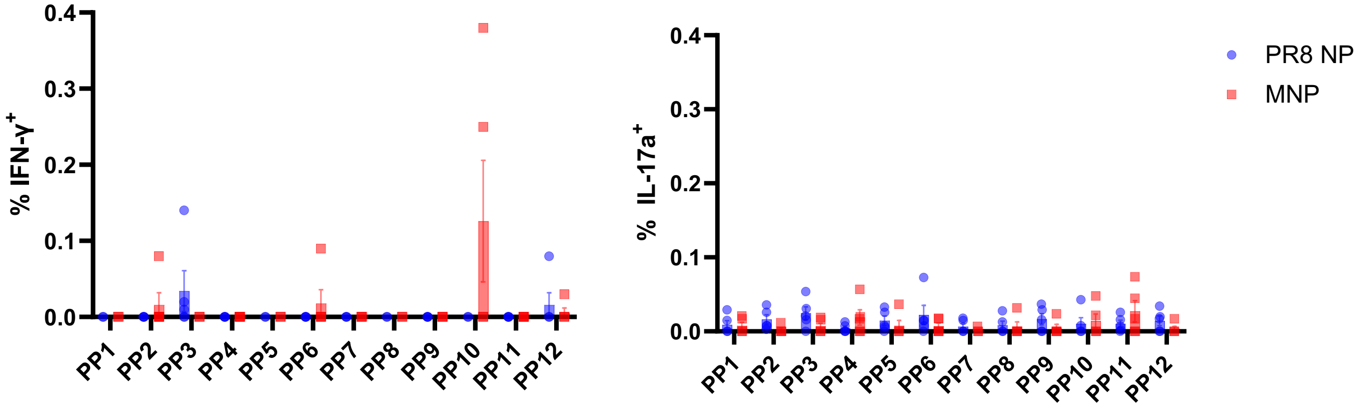

B

Epitope Prediction Ranking	PR8 NP	MNP	CA04 NP
1	NP366	NP366	NP366
2	P10	P1	P1
3	P11	P11	P2
4	P3	P3	P3
5	P4	P4	P4
6	P12	P12	P5
7	-	P14	P6
8	-	-	P7
9	-	-	P8
10	P13	-	P9

CD8 T cells

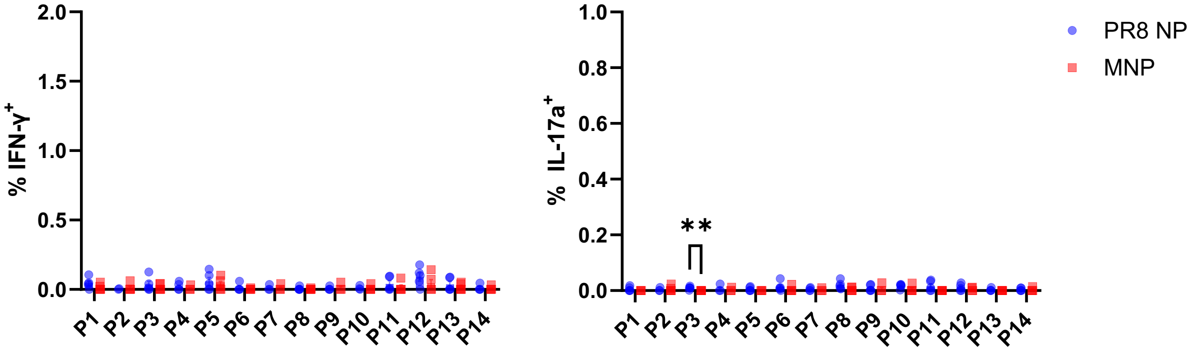

Supplemental Figure 3.

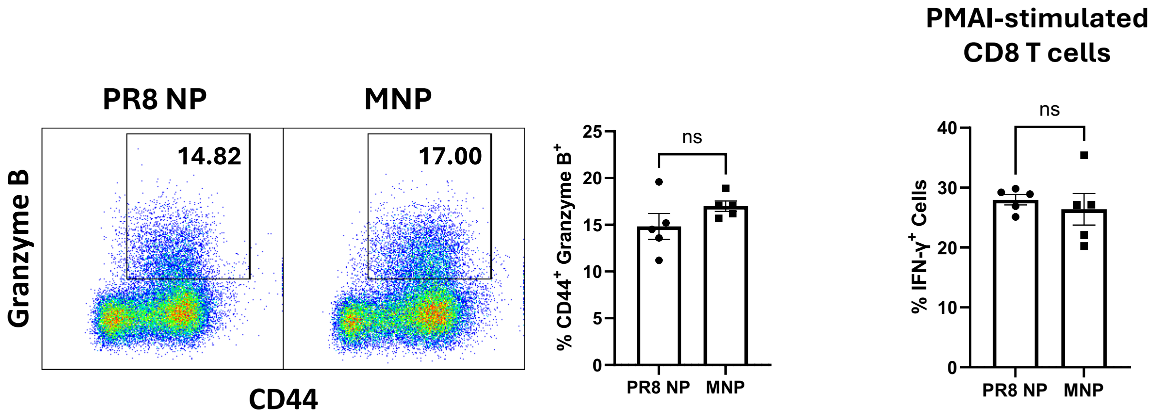

Supplemental Figure 4.

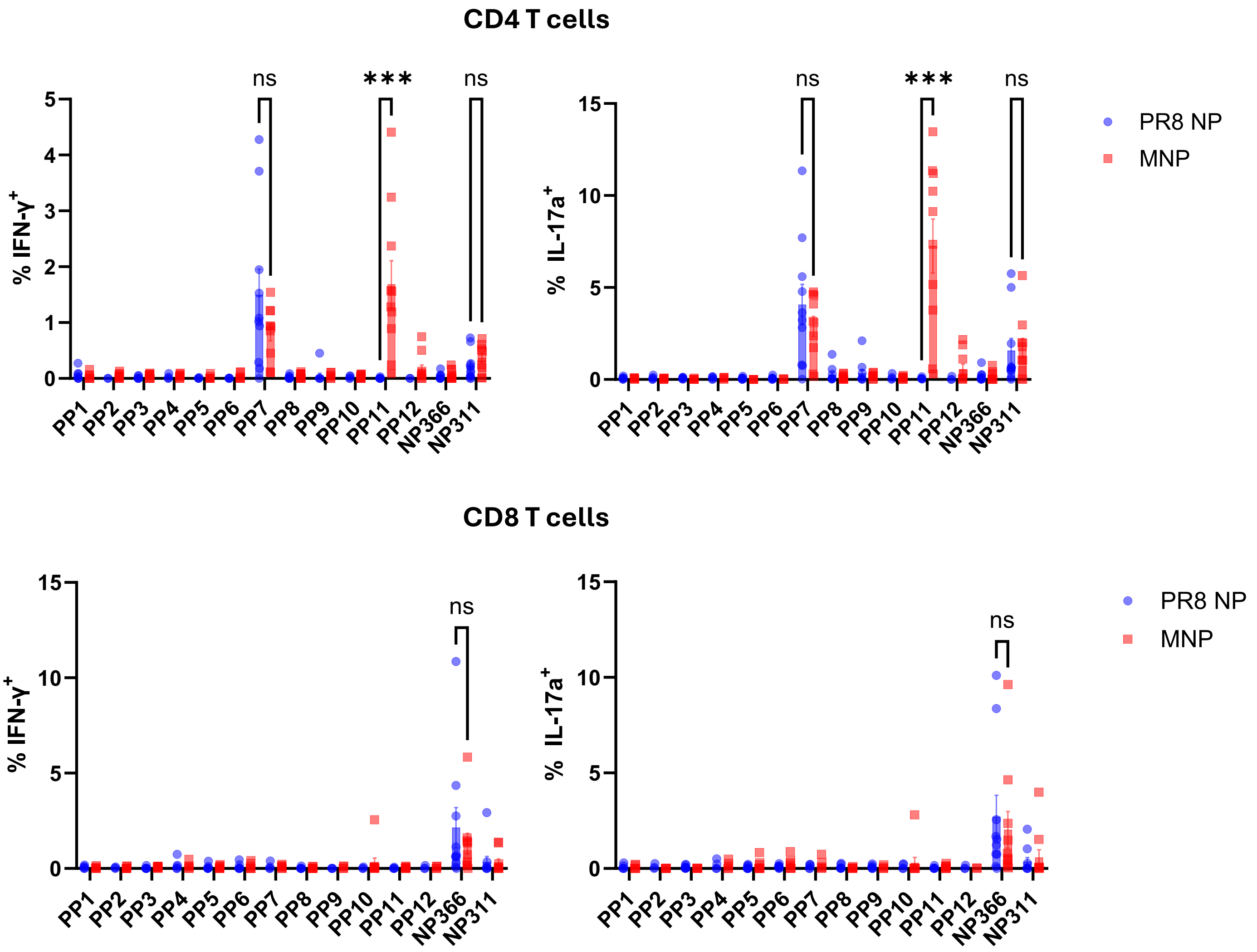

Supplemental Figure 5.

Epitope Prediction Ranking	PR8 NP	MNP	CA04 NP
1	NP366	NP366	NP366
2	P10	P1	P1
3	P11	P11	P2
4	P3	P3	P3
5	P4	P4	P4
6	P12	P12	P5
7	-	P14	P6
8	-	-	P7
9	-	-	P8
10	P13	-	P9

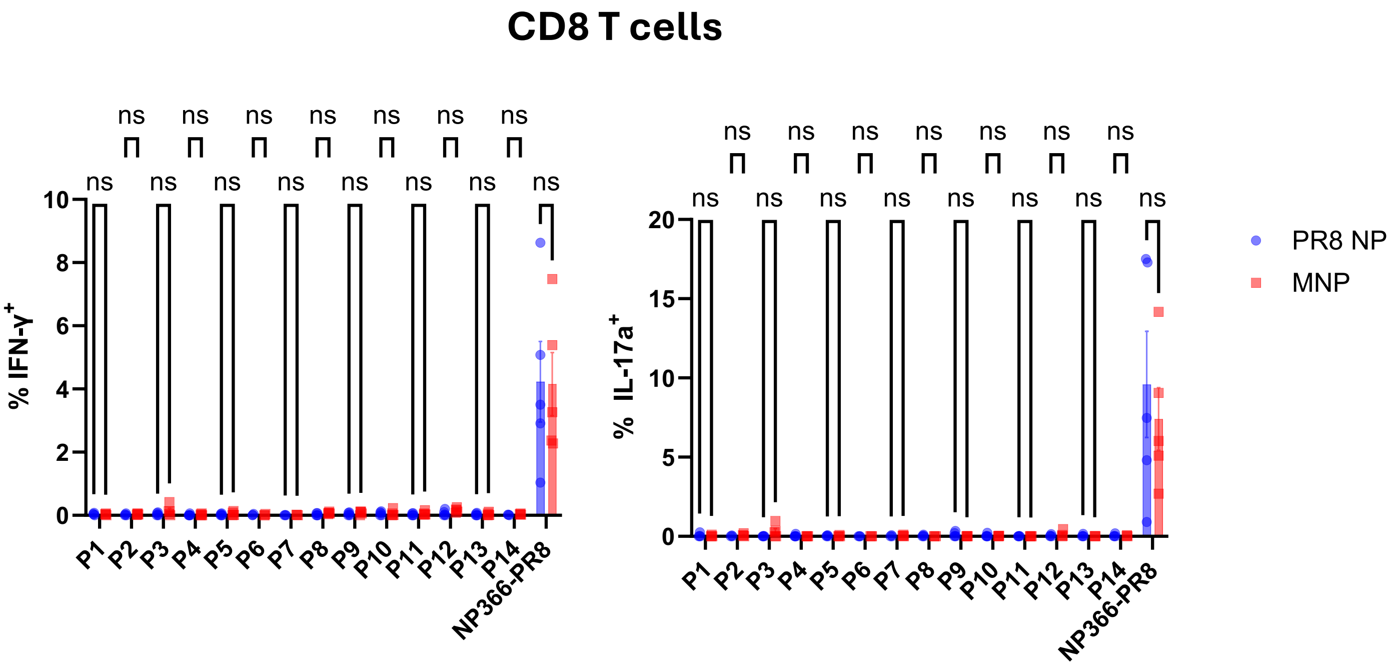

Supplement: Supplemental figures — Figures S1 to S5. [file jvi.00867-24-s0001.pdf]
