# Supplementary material for: Diversifying T-cell responses: safeguarding against pandemic influenza with mosaic nucleoprotein
Source: J Virol. 2025 Feb 3;99(3):e00867-24. doi: 10.1128/jvi.00867-24 (PMC11915837; doi:10.1128/jvi.00867-24)
Supplement: Supplemental legends — Legends for Fig. S1 to S5. [file jvi.00867-24-s0002.docx]

**Supplemental Figure Legends**

**Figure S1. Epitope coverage in MNP and amino acid sequence alignment of the nucleoprotein (NP) from six different IAV strains. (A)** Sequence coverage for all unique 9-mer epitopes in 7,422 influenza NP was assessed by comparing them to the MNP sequence. **(B)** Sequences of NP from six different Influenza A virus strains were aligned with mosaic NP using Clustal Omega (https://www.ebi.ac.uk/jdispatcher/msa/clustalo) and visualized by Jalview software (v.2.11.3.2).

**Figure S2. Detection of peptide-pool-specific CD4 or CD8 T cells in the lungs after challenge.** Mice were vaccinated with PR8 NP or mosaic NP (MNP) formulated in ADJ+GLA. Vaccinated mice were challenged with IAV-CA04, and on the 6^th^ day after challenge, single cell suspensions of lung tissues were stimulated with IAV-CAO4 NP peptide pools (A) or the synthetic peptides derived from top 10 predicted epitopes for CD8 T cells (B); IFN-γ and/or IL-17- producing CD4 or CD8 T cells were quantified by intracellular cytokine staining.

**Figure S3. Detection of cytotoxic CD8 T cells in the lungs.** Cohorts of mice vaccinated with PR8 NP or mosaic NP (MNP) were challenged with IAV-CAO4. On the 6^th^ day after challenge, Granzyme B-expressing CD8 T cells were quantified by flow cytometry. Lung cells were stimulated with PMAI and IFN-γ-producing CD8 T cells were quantified using intracellular cytokine staining.

**Figure S4. Detection of peptide-specific CD4 or CD8 T cells in the lungs after vaccination.** Cohorts of C57BL/6 mice were vaccinated with PR8 NP or mosaic NP (MNP) formulated in Adjuplex+GLA. On the 8^th^ day after booster vaccinations, lung cells were stimulated with the indicated peptides, and CD4 or CD8 T cells expressing IFN-γ and/or IL-17 were quantified by intracellular cytokine staining.

**Figure S5. Detection of peptide-specific CD4 and CD8 T cells in the lungs after vaccination.** Cohorts of C57BL/6 mice were vaccinated with PR8 NP or mosaic NP (MNP) formulated in Adjuplex + GLA. On the 8th day after booster vaccination, lung cells were stimulated with peptides corresponding to high-ranking epitope sequences predicted by the MHC Class I prediction tool, and CD4 and CD8 T cells expressing IFN-γ and/or IL-17 were quantified by intracellular cytokine staining.
