# Supplementary material for: Diversifying T-cell responses: safeguarding against pandemic influenza with mosaic nucleoprotein
Source: J Virol. 2025 Feb 3;99(3):e00867-24. doi: 10.1128/jvi.00867-24 (PMC11915837; doi:10.1128/jvi.00867-24)
Supplement: Table S1 — Antibodies and peptides. [file jvi.00867-24-s0003.docx]

Supplemental Table 1. List of antibodies and peptides used in the manuscript

| **Reagent or Resource** |  | **Source** | **Identifier** |
| --- | --- | --- | --- |
| **Antibody** | **Dilution factor** |  |  |
| Rat anti-mouse CD8α-BUV395-conjugated (53-6.7) | 1:200 | BD Biosciences | 563786 |
| Rat anti-mouse CD4-BUV496-conjugated (GK1.5) | 1:200 |  | 564667 |
| Rat anti-mouse CD44-BV510-conjugated (IM7) | 1:200 |  | 563144 |
| Rat anti-mouse IFN-γ-Alexa Fluor 700-conjugated (XMG1.2) | 1:300 |  | 557998 |
| Rat anti-mouse IL-17A-FITC-conjugated (TC11-18H10.1) | 1:200 | Biolegend | 506908 |
| Rat anti-human/mouse Granzyme B-PE-Cy7-conjugated (QA18A28) | 1:50 |  | 396410 |
| Rat anti-mouse CD44-FITC-conjugated (IM7) | 1:200 | BD Bioscience | 553133 |
| Rat anti-mouse IL-17A-PE-conjugated (TC11-18H10) | 1:200 |  | 561020 |
| BV421-conjugated I-Ab tetramers bearing the NP peptide NP311 (QVYSLIRPNENPAHK) | 1:150 | NIH Tetramer Core Facility at Emory University | N/A |
| PE-conjugated I-Ab tetramers bearing the NP peptide NP311 (QVYSLIRPNENPAHK) | 1:150 |  | N/A |
| APC-conjugated-H2-Db tetramers bearing the NP peptide NP366 (ASNENMETM) | 1:150 |  | N/A |
| **Peptides** | **Sequence** |  |  |
| NP366-PR8 | ASNENMETM | ProImmune |  |
| NP311-PR8 | QVYSLIRPNENPAHK |  |  |
| NP366-Aichi | ASNENMDAM | GenScript |  |
| NP366-CA04 | ASNENVETM |  |  |
| P1 | RLIQNSITI |  |  |
| P2 | VGIDPFKLL |  |  |
| P3 | ATYQRTRAL |  |  |
| P4 | YSLVGIDPF |  |  |
| P5 | VAYERMCNI |  |  |
| P6 | KLLQNSQVV |  |  |
| P7 | TMDSNTLEL |  |  |
| P8 | HIMIWHSNL |  |  |
| P9 | SSFIRGKKV |  |  |
| P10 | RLIQNSLTI |  |  |
| P11 | VGIDPFRLL |  |  |
| P12 | IAYERMCNI |  |  |
| P13 | HMMIWHSNL |  |  |
| P14 | SSFIRGTRV |  |  |
| NP75-89 | RNKYLEEHPSAGKDP |  |  |
| NP263-277 | ALILRGSVAHKSCLP |  |  |
| NP276-290-PR8 | LPACVYGPAVASGYD |  |  |
| NP311-325-CA04 | QVVSLMRPNENPAHK |  |  |
| NP417-431-MNP | NLPFERATIMAAFTG |  |  |
| **CA04 peptide array** |  |  |  |
| 1 of 122 | 1-MASQGTKRSYEQMET-15 | BEI Resources | LS1884 |
| 2 of 122 | 5-GTKRSYEQMETGGER-19 |  | LS1885 |
| 3 of 122 | 9-SYEQMETGGERQDAT-23 |  | LS1886 |
| 4 of 122 | 13-METGGERQDATEIRA-27 |  | LS1887 |
| 5 of 122 | 17-GERQDATEIRASVGR-31 |  | LS1888 |
| 6 of 122 | 21-DATEIRASVGRMIGG-35 |  | LS1889 |
| 7 of 122 | 25-IRASVGRMIGGIGRF-39 |  | LS1890 |
| 8 of 122 | 29-VGRMIGGIGRFYIQM-43 |  | LS1891 |
| 9 of 122 | 33-IGGIGRFYIQMCTEL-47 |  | LS1892 |
| 10 of 122 | 37-GRFYIQMCTELKLSD-51 |  | A4748 |
| 11 of 122 | 41-IQMCTELKLSDYDGR-55 |  | A4788 |
| 12 of 122 | 45-TELKLSDYDGRLIQN-59 |  | LS1895 |
| 13 of 122 | 49-LSDYDGRLIQNSITI-63 |  | LS1896 |
| 14 of 122 | 53-DGRLIQNSITIERMV-67 |  | LS1897 |
| 15 of 122 | 57-IQNSITIERMVLSAF-71 |  | LS1898 |
| 16 of 122 | 61-ITIERMVLSAFDERR-75 |  | LS1899 |
| 17 of 122 | 65-RMVLSAFDERRNKYL-79 |  | LS1900 |
| 18 of 122 | 69-SAFDERRNKYLEEHPS-84 |  | LS1901 |
| 19 of 122 | 73-ERRNKYLEEHPSAGK-87 |  | LS1902 |
| 20 of 122 | 77-KYLEEHPSAGKDPKK-91 |  | LS1903 |
| 21 of 122 | 81-EHPSAGKDPKKTGGPI-96 |  | LS1904 |
| 22 of 122 | 85-AGKDPKKTGGPIYRR-99 |  | LS1905 |
| 23 of 122 | 89-PKKTGGPIYRRVDGK-103 |  | LS1906 |
| 24 of 122 | 93-GGPIYRRVDGKWMRE-107 |  | LS1907 |
| 25 of 122 | 97-YRRVDGKWMRELILY-111 |  | LS1908 |
| 26 of 122 | 101-DGKWMRELILYDKEE-115 |  | LS1909 |
| 27 of 122 | 105-MRELILYDKEEIRRV-119 |  | LS1910 |
| 28 of 122 | 109-ILYDKEEIRRVWRQA-123 |  | LS1911 |
| 29 of 122 | 113-KEEIRRVWRQANNGE-127 |  | LS1912 |
| 30 of 122 | 117-RRVWRQANNGEDATA-131 |  | LS1913 |
| 31 of 122 | 121-RQANNGEDATAGLTH-135 |  | LS1914 |
| 32 of 122 | 125-NGEDATAGLTHIMIW-139 |  | LS1915 |
| 33 of 122 | 129-ATAGLTHIMIWHSNL-143 |  | LS1916 |
| 34 of 122 | 133-LTHIMIWHSNLNDAT-147 |  | LS1917 |
| 35 of 122 | 137-MIWHSNLNDATYQRT-151 |  | LS1918 |
| 36 of 122 | 141-SNLNDATYQRTRALV-155 |  | LS1919 |
| 37 of 122 | 145-DATYQRTRALVRTGM-159 |  | LS1920 |
| 38 of 122 | 150-RTRALVRTGMDPRM-163 |  | LS1921 |
| 39 of 122 | 153-ALVRTGMDPRMCSLM-167 |  | LS1922 |
| 40 of 122 | 157-TGMDPRMCSLMQGST-171 |  | LS1923 |
| 41 of 122 | 161-PRMCSLMQGSTLPRR-175 |  | LS1924 |
| 42 of 122 | 165-SLMQGSTLPRRSGAA-179 |  | LS1925 |
| 43 of 122 | 169-GSTLPRRSGAAGAAV-183 |  | LS1926 |
| 44 of 122 | 173-PRRSGAAGAAVKGVG-187 |  | LS1927 |
| 45 of 122 | 177-GAAGAAVKGVGTIAM-191 |  | LS1928 |
| 46 of 122 | 181-AAVKGVGTIAMELIR-195 |  | LS1929 |
| 47 of 122 | 185-GVGTIAMELIRMIKR-199 |  | LS1930 |
| 48 of 122 | 189-IAMELIRMIKRGIND-203 |  | LS1931-2 |
| 49 of 122 | 193-LIRMIKRGINDRNFW-207 |  | LS1932 |
| 50 of 122 | 197-IKRGINDRNFWRGEN-211 |  | LS1933 |
| 51 of 122 | 201-INDRNFWRGENGRRT-215 |  | LS1934 |
| 52 of 122 | 205-NFWRGENGRRTRVAY-219 |  | LS1935 |
| 53 of 122 | 209-GENGRRTRVAYERMC-223 |  | LS1936 |
| 54 of 122 | 213-RRTRVAYERMCNILK-227 |  | LS1937 |
| 55 of 122 | 217-VAYERMCNILKGKFQ-231 |  | LS1938 |
| 56 of 122 | 221-RMCNILKGKFQTAAQ-235 |  | LS1939 |
| 57 of 122 | 225-ILKGKFQTAAQRAMM-239 |  | LS1940 |
| 58 of 122 | 229-KFQTAAQRAMMDQVR-243 |  | LS1941 |
| 59 of 122 | 233-AAQRAMMDQVRESRN-247 |  | LS1942 |
| 60 of 122 | 237-AMMDQVRESRNPGNA-251 |  | LS1943 |
| 61 of 122 | 242-VRESRNPGNAEIED-255 |  | LS1944 |
| 62 of 122 | 245-SRNPGNAEIEDLIFL-259 |  | LS1945 |
| 63 of 122 | 249-GNAEIEDLIFLARSA-263 |  | LS1946 |
| 64 of 122 | 253-IEDLIFLARSALILR-267 |  | LS1947 |
| 65 of 122 | 257-IFLARSALILRGSVA-271 |  | LS1948 |
| 66 of 122 | 261-RSALILRGSVAHKSC-275 |  | LS1949-2 |
| 67 of 122 | 265-ILRGSVAHKSCLPAC-279 |  | LS1950 |
| 68 of 122 | 269-SVAHKSCLPACVYGL-283 |  | LS1951 |
| 69 of 122 | 273-KSCLPACVYGLAVAS-287 |  | LS1952 |
| 70 of 122 | 277-PACVYGLAVASGHDF-291 |  | LS1953 |
| 71 of 122 | 281-YGLAVASGHDFEREG-295 |  | LS1954 |
| 72 of 122 | 285-VASGHDFEREGYSLV-299 |  | LS1955 |
| 73 of 122 | 289-HDFEREGYSLVGIDPF-304 |  | LS1956 |
| 74 of 122 | 293-REGYSLVGIDPFKLL-307 |  | LS1957 |
| 75 of 122 | 297-SLVGIDPFKLLQNSQ-311 |  | LS1958 |
| 76 of 122 | 301-IDPFKLLQNSQVVSL-315 |  | LS1959 |
| 77 of 122 | 305-KLLQNSQVVSLMRPN-319 |  | LS1960 |
| 78 of 122 | 309-NSQVVSLMRPNENPA-323 |  | LS1961 |
| 79 of 122 | 313-VSLMRPNENPAHKSQ-327 |  | LS1962 |
| 80 of 122 | 317-RPNENPAHKSQLVWM-331 |  | LS1963 |
| 81 of 122 | 321-NPAHKSQLVWMACHS-335 |  | LS1964 |
| 82 of 122 | 325-KSQLVWMACHSAAFE-339 |  | LS1965 |
| 83 of 122 | 329-VWMACHSAAFEDLRV-343 |  | LS1966 |
| 84 of 122 | 333-CHSAAFEDLRVSSFI-347 |  | LS1967 |
| 85 of 122 | 337-AFEDLRVSSFIRGKK-351 |  | LS1968 |
| 86 of 122 | 341-LRVSSFIRGKKVIPR-355 |  | LS1969 |
| 87 of 122 | 345-SFIRGKKVIPRGKLS-359 |  | LS1970 |
| 88 of 122 | 349-GKKVIPRGKLSTRGV-363 |  | LS1971 |
| 89 of 122 | 353-IPRGKLSTRGVQIAS-367 |  | LS1972 |
| 90 of 122 | 357-KLSTRGVQIASNENV-371 |  | LS1973 |
| 91 of 122 | 361-RGVQIASNENVETMD-375 |  | LS1974 |
| 92 of 122 | 365-IASNENVETMDSNTL-379 |  | 2091-9/14 |
| 93 of 122 | 369-ENVETMDSNTLELRS-383 |  | LS1976 |
| 94 of 122 | 373-TMDSNTLELRSRYWA-387 |  | LS1977 |
| 95 of 122 | 377-NTLELRSRYWAIRTR-391 |  | LS1978 |
| 96 of 122 | 381-LRSRYWAIRTRSGGN-395 |  | LS1979 |
| 97 of 122 | 385-YWAIRTRSGGNTNQQ-399 |  | LS1980 |
| 98 of 122 | 389-RTRSGGNTNQQKASA-403 |  | LS1981 |
| 99 of 122 | 393-GGNTNQQKASAGQIS-407 |  | LS1982 |
| 100 of 122 | 397-NQQKASAGQISVQPT-411 |  | LS1983 |
| 101 of 122 | 401-ASAGQISVQPTFSVQ-415 |  | LS1984 |
| 102 of 122 | 406-ISVQPTFSVQRNLPF-420 |  | LS1985 |
| 103 of 122 | 410-PTFSVQRNLPFERA-423 |  | LS1986 |
| 104 of 122 | 413-SVQRNLPFERATVMA-427 |  | LS1987 |
| 105 of 122 | 417-NLPFERATVMAAFSG-431 |  | LS1988 |
| 106 of 122 | 421-ERATVMAAFSGNNEG-435 |  | LS1989 |
| 107 of 122 | 425-VMAAFSGNNEGRTSD-439 |  | LS1990 |
| 108 of 122 | 429-FSGNNEGRTSDMRTE-443 |  | LS1991 |
| 109 of 122 | 433-NEGRTSDMRTEVIRM-447 |  | LS1992 |
| 110 of 122 | 437-TSDMRTEVIRMMESA-451 |  | LS1993 |
| 111 of 122 | 441-RTEVIRMMESAKPED-455 |  | LS1994 |
| 112 of 122 | 445-IRMMESAKPEDLSFQ-459 |  | LS1995 |
| 113 of 122 | 449-ESAKPEDLSFQGRGV-463 |  | LS1996 |
| 114 of 122 | 453-PEDLSFQGRGVFELS-467 |  | LS1997 |
| 115 of 122 | 457-SFQGRGVFELSDEKA-471 |  | LS1998 |
| 116 of 122 | 461-RGVFELSDEKATNPI-475 |  | LS1999 |
| 117 of 122 | 465-ELSDEKATNPIVPSF-479 |  | LS2000 |
| 118 of 122 | 469-EKATNPIVPSFDMSN-483 |  | LS2001 |
| 119 of 122 | 473-NPIVPSFDMSNEGSY-487 |  | LS2002 |
| 120 of 122 | 477-PSFDMSNEGSYFFGD-491 |  | LS2003 |
| 121 of 122 | 481-MSNEGSYFFGDNAEE-495 |  | LS2004 |
| 122 of 122 | 485-GSYFFGDNAEEYDS-498 |  | LS2005 |
